# Effects of artificial ultraviolet B radiation on the macrophyte *Lemna minor:* a conceptual study for toxicity pathway characterization

**DOI:** 10.1007/s00425-020-03482-3

**Published:** 2020-10-14

**Authors:** Li Xie, Knut Asbjørn Solhaug, You Song, Bjørn Johnsen, Jorunn Elisabeth Olsen, Knut Erik Tollefsen

**Affiliations:** 1 Section of Ecotoxicology and Risk Assessment, Norwegian Institute for Water Research (NIVA), Gaustadalléen 21, 0349 Oslo, Norway; 2 Faculty of Environmental Sciences and Natural Resource Management, Norwegian University of Life Sciences (NMBU), P.O. Box 5003, 1432 Ås, Norway; 3 Centre for Environmental Radioactivity, Norwegian University of Life Sciences (NMBU), Post Box 5003, 1432 Ås, Norway; 4 Norwegian Radiation and Nuclear Safety Authority (DSA), 1361 Østerås, Norway; 5 Faculty of Biosciences, Institute of Plant Sciences, Norwegian University of Life Sciences (NMBU), P.O. Box 5003, 1432 Ås, Norway

**Keywords:** Aquatic plant, Gene expression, Mode of action, Non-ionizing radiation, Toxicity pathway

## Abstract

**Main conclusion:**

UVB radiation caused irradiance-dependent and target-specific responses in non-UVB acclimated *Lemna minor*. Conceptual toxicity pathways were developed to propose causal relationships between UVB-mediated effects at multiple levels of biological organisation.

**Abstract:**

Macrophytes inhabit waterways around the world and are used in hydroponics or aquaponics for different purposes such as feed and wastewater treatment and are thus exposed to elevated levels of UVB from natural and artificial sources. Although high UVB levels are harmful to macrophytes, mechanistic understanding of irradiance-dependent effects and associated modes of action in non-UVB acclimated plants still remains low. The present study was conducted to characterise the irradiance-dependent mechanisms of UVB leading to growth inhibition in *Lemna minor* as an aquatic macrophyte model. The *L. minor* were continuously exposed to UVB (0.008–4.2 W m^−2^) and constant UVA (4 W m^−2^) and photosynthetically active radiation, PAR (80 µmol m^−2^ s^−1^) for 7 days. A suite of bioassays was deployed to assess effects on oxidative stress, photosynthesis, DNA damage, and transcription of antioxidant biosynthesis, DNA repair, programmed cell death, pigment metabolism and respiration. The results showed that UVB triggered both irradiance-dependent and target-specific effects at multiple levels of biological organization, whereas exposure to UVA alone did not cause any effects. Inhibition of photosystem II and induction of carotenoids were observed at 0.23 W m^−2^, whereas growth inhibition, excessive reactive oxygen species, lipid peroxidation, cyclobutane pyrimidine dimer formation, mitochondrial membrane potential reduction and chlorophyll depletion were observed at 0.5–1 W m^−2^. Relationships between responses at different levels of biological organization were used to establish a putative network of toxicity pathways to improve our understanding of UVB effects in aquatic macrophytes under continuous UVB exposures. Additional studies under natural illuminations were proposed to assess whether these putative toxicity pathways may also be relevant for more ecologically relevant exposure scenarios.

**Electronic supplementary material:**

The online version of this article (10.1007/s00425-020-03482-3) contains supplementary material, which is available to authorized users.

## Introduction

Ultraviolet radiation (UVR) is an important type of non-ionizing solar radiation and is known to cause detrimental, positive and regulatory effects in different organisms. UV-mediated effects are highly wavelength dependent, the ratio of UVR to photosynthetically active irradiation (PAR) and the organisms’ baseline tolerance and susceptibility for UVR (Bornman et al. 2019; Williamson et al. 2019). As a part of the electromagnetic spectrum, UVR comprises about 8–9% of the solar irradiance at the top of the atmosphere (Whitehead et al. 2000). The solar UVR reaching the ground can be divided into UVB (280–315 nm) and UVA (315–400 nm), where solar UVB is partly absorbed by the ozone layer, whereas UVA is unaffected by ozone. In the past few decades, depletion of atmospheric ozone has in particular led to concern for enhanced UV radiation at earth’s surface due to reduction in the UV absorption properties of ozone. Although the average of total globally ozone in recent years (2014–2017) remains only 2.2% below the average of the 1964–1980 period (Bais et al. 2019), the biological impact of UVB on organisms is a still a major scientific topic as it has been proposed by the Environmental Effects Assessment Panel (EEAP) that climate change makes the ecosystems more vulnerable to effects of UV radiation on a number of biotic and non-biotic targets (Bernhard et al. 2020). Artificial UVB is also being increasingly used for disinfection and disease control in a number of technical applications (Stouvenakers et al. 2019). Undoubtedly, it is still important to improve the mechanistic understanding of the irradiance-dependent effects of UVB on organisms.

It is recognized today that UVR affects plant growth by regulating key physiological processes such as (among others) auxin and gibberellin production, photosynthesis, pigment formation and integrity (Rozema et al. 1997; Roro et al. 2017). Exposure to low levels of UVR can induce natural defense mechanisms in plants to prevent damage to plant cells, including biosynthesis and accumulation of UV-absorbing or screening compounds (Cockell and Knowland 1999). Enhanced UV, and in particular UVB, can also induce adverse effects in plants, typically under a high UVB to PAR ratios and elevated UVB levels (Teramura and Sullivan 1994). In general, elevated UVB can cause damage to cell membranes and DNA, lead to the generation of reactive oxygen species (ROS) and ultimately disrupt cellular homeostasis (Hideg et al. 1994). Among the many biological effects observed, reduced photosynthesis rates and subsequently reduced primary production are the most severe effects reported. Despite the increasing amount of data on the effects of UVR on terrestrial plants, there is limited knowledge on the effects of UVR on aquatic plants and the causal relationships between effects occurring at different levels of biological organization.

In freshwater ecosystems, the common duckweed *L. minor* is widely distributed and an important species in the aquatic food webs (Talukdar et al. 2013). Due to its rapid reproduction rate in limited space, *L. minor* is especially suitable for use in short-term laboratory studies (Radić et al. 2011) and field monitoring approaches (Coors et al. 2006). Among the freshwater primary producers, *L. minor* as a floating macrophyte may have higher probability for being exposed to elevated UVB irradiance as they live at the water surface where irradiation is high. Farooq et al. (2000) have also proposed that *L*. minor is sensitive to artificial UVB and suggested it used as a sentinel species for impact assessment of enhanced UVB irradiance under standardized laboratory conditions. In addition, *L. minor* has gained popularity in aquaculture and wastewater treatment processes, either as a supplemental protein resource for feeding domestic animals and fish (Ekperusi et al. 2019), or as a biological filter to reduce harmful substances (Velichkova and Sirakov 2013). In this respect, aquatic (and terrestrial) plants may be cultivated under modified or artificial light conditions, potentially with elevated UVB irradiance for phytopathogen control (Suthaparan et al. 2017; Rivas-García et al. 2020). In such cases, plants may temporarily receive high UVB irradiance without prior acclimation, a condition which might induce physiological changes in the plants that affect biomass production. However, the knowledge of how molecular and cellular changes induced by UVB affect growth and development in plants is still poorly developed and thus warrants further investigation.

The objective of the present study was to characterize the biological impacts of elevated UVB levels in *L. minor* under standard aquatic toxicity test conditions and investigate the potential toxic and/or regulatory mechanisms across different levels of biological organization. In this respect, non-UVB-acclimated *L. minor* was exposed for 7 days to elevated UVB irradiances (0.23–4.2 W m^−2^) together with a constant UVA irradiance (4 W m^−2^) and PAR (80 µmol m^−2^ s^−1^, approx.) under growth conditions defined by the OECD Test guideline 221 (OECD 2006). Continuous artificial illumination with low UVA and PAR was used to reduce the influence of light- and dark-driven repair mechanisms to characterise (and accentuate) the adverse effects and modes of action (MoAs) of UVB in this aquatic plant. A number of biological endpoints were determined to assess the effects of UVB on reactive oxygen species (ROS) formation, lipid peroxidation (LPO), mitochondrial membrane potential (MMP), photosynthetic efficiency (PSII performance), pigment content (chlorophyll *a*, chlorophyll *b* and total carotenoids) and DNA damage (formation of cyclobutane pyrimidine dimers, CPD). Expression of biomarker genes representative of different responses was assessed to provide in-depth characterisation of the toxicity and regulatory signalling pathways triggered. Correlational analysis was conducted to identify potential relationships between the effects of UVB at different biological levels of organization to facilitate proposing the most relevant toxicity pathways for the stressor.

## Materials and methods

### Test organism and culture conditions

The test species *Lemna minor* L. (strain ID: 5544, Rutgers Duckweed Stock Cooperative) was obtained from Ghent University, Belgium. After disinfection by 0.5% NaOCl (v/v), the *L. minor* cultures were maintained in Swedish Standard (SIS) medium (Moody and Miller 2005) in a growth chamber at 24 °C under continuous white light from fluorescent lamps (L 36W/77-G13, Centra Osram, Berlin, German) with a PAR of 80 ± 5 µmol m^−2^ s^−1^ following the OECD TG221 guideline (OECD 2006), with stock thalli sub-cultured twice a week. The combinations of UVB, UVA and PAR were carefully chosen to assess the effects of different UVB irradiances under artificial conditions with relatively low photosynthetic photon flux density rather than mimicking growth conditions in nature. The irradiance was measured with a LI-COR quantum sensor Model LI-190 (Lincoln, NE, USA) connected to a LI-COR LI-250 photometer unit.

### UVR exposure

A range of different UVB irradiances were used together with a constant low UVA irradiance, as UVA radiation is a factor for photorepair in plants (Allen et al. 1998). The studies were conducted in a custom-made UV exposure chamber consisting of a Multitron-Pro incubator (Infors HT, Bottmingen, Switzerland), equipped with fluorescent UVA tubes (UVA 36W/78, Centra Osram, peak emission approximately 330 nm) and PL‐UVB tubes (L 36W/UVB UV6, Waldmann, Villingen-Schwenningen, Germany, peak emission approximately 280 nm) and 80 µmol m^−2^ s^−1^ PAR. Twelve healthy *L. minor* fronds per beaker (*n* = 4 beakers) in 40 mL SIS medium were exposed continuously to different UVB irradiances (UVB 0.23–4.2 W m^−2^, UVA 4.56 ± 0.31 W m^−2^, PAR 81.1 ± 0.38 µmol m^−2^ s^−1^), UVA-control, UVA-CT (UVB 0.008 W m^−2^, UVA 4.25 ± 0.47 W m^−2^, PAR 79.6 ± 1.7 µmol m^−2^ s^−1^) and a non-UV control, non-UV-CT (PAR 79.9 ± 0.4 µmol m^−2^ s^−1^) for 7 days (Table 1) at the same conditions as during the preculture. As UVB tubes also emit UVA radiation, the UVA irradiance was maintained as low as possible to reduce the effects on *L. minor*. During exposure, the UVA control was covered with pre-burned (24 h exposed to 1 W m^−2^ UVB) polyester foil (0.175 mm, Nordbergs Tekniska AB, Vallentuna, Sweden) to completely block UVC and most UVB radiation (wavelength < 315 nm). The treatments groups were covered with pre-burned (24 h exposed to 1 W m^−2^ UVB) cellulose acetate (0.13 mm, Jürgen Rachow, Hamburg, Germany) to filter out wavelengths below 290 nm (UVC and the shortest UVB wavelengths). The exclusion of unwanted shortwave UV, by applying polyester and cellulose acetate filters to the UVB and UVA tubes, was confirmed by measurements by the Norwegian Radiation and Nuclear Safety Authority (DSA, Oslo, Norway), applying a calibrated Bentham DTM300 high-resolution scanning spectroradiometer (Bentham DTM 300, Bentham Instruments Ltd, Reading, UK) and presented in Suppl. Fig. S1. Irradiances of UVA, UVB and PAR to *L. minor* were determined before and at the end of the exposure experiments with a SpectroSense 2+ filter radiometer (Skye Instruments Ltd, Llandrindod Wells, UK), calibrated against the Bentham spectroradiometer at DSA. The irradiance of UVA, UVB and PAR as well as water quality conditions such as pH and temperature was monitored regularly throughout the study (Suppl. Fig. S2 and Fig. S3). No significant changes in UVB, UVA, PAR and temperature were observed during the study. The pH increased by maximum of 10% in the culture medium in all groups including controls (from pH of 6.52 to 7.11) after exposure. However, no significant difference in pH was observed between the non-UV control and the treatments after 7 days’ exposure.

**Table 1:** Irradiances of photosynthetically active radiation (PAR), UVA and UVB in the 7-day exposure study with *Lemna minor*

Exposure group	PAR (µmol m^−2^ s^−1^)	UVA irradiance (W m^−2^)	UVB irradiance (W m^−2^)
Non-UV-CT	79.9 ± 0.4	0	0
UVA-CT	79.6 ± 1.7	4.253 ± 0.472	0.008 ± 0.002
UVB-0.25	81.5 ± 3.1	4.575 ± 0.387	0.232 ± 0.033
UVB-0.5	81.4 ± 1.8	4.333 ± 0.414	0.486 ± 0.041
UVB-0.1	80.7 ± 2.5	4.614 ± 0.496	1.075 ± 0.084
UVB-2	81.3 ± 2.1	4.866 ± 0.532	2.044 ± 0.156
UVB-4	80.7 ± 1.6	4.781 ± 0.463	4.181 ± 0.233

The values are mean of 4 repeated measurements ± standard error (SE)

### Growth responses

The recorded growth parameters of *L. minor* are traditional toxicological and regulatory-relevant endpoints that have relevance for maintaining a healthy population of plants. The frond number (FN) was scored at the start and end of experiments, and the growth inhibition was calculated as described in the OECD Test guideline 221 *Lemna* sp. growth Inhibition Test (OECD 2006). The test was considered valid when the growth rate measured as frond number (FN) in the control groups was higher than 0.275 day^−1^ (OECD 2006). For measurement of frond area (FA), the area of individual floating frond was determined optically by a digital camera (FinePix S2500HD, Fujifilm, Tokyo, Japan) using a floating scale bar. The frond area in each photograph was analysed using the Image-J software program version 1.48 (National Institutes of Health, MD, USA). To measure the dry mass (DM), fronds were dried in an oven at 70 °C until constant weight was obtained.

### Cyclobutane pyrimidine dimers assay

Cyclobutane pyrimidine dimers (CPD), as a marker of UV-induced direct DNA damage, were determined by an Oxiselect™ UV-Induced DNA Damage ELISA kit (STA-322; Cell Biolabs, San Diego, CA, USA) following the vendor`s instructions. After 7 days of exposure, DNA was extracted from *L. minor* using the DNeasy Plant Mini Kit (Qiagen, Hilden, Germany) and converted to single-stranded DNA (ssDNA) by incubation at 95 °C for 10 min and rapidly cooled on ice for 10 min. Using the Oxiselect ELISA kit, the CPDs were quantified by the binding of the DNA to an anti-CPD antibody followed by a Horse radish peroxidase (HRP)-conjugated secondary antibody. The CPD levels were quantified with a standard curve derived from a series of diluted CPD-DNA standards from the Oxiselect ELISA kit (no. 232203).

### Reactive oxygen species (ROS) assays

The cellular reactive oxygen species level in *L. minor* was quantified using the 2′,7′-dichlorofluorescein diacetate (H_2_DCFDA) assay (Molecular Probes Inc., Eugene, OR, USA), as originally developed by Razinger et al. (2010) and further modified for *L. minor* by Xie et al. (2018). When binding to ROS (predominantly hydrogen peroxide), non-fluorescent H_2_DCFDA will be oxidized to the highly fluorescent 2′,7′-dichlorofluorescein (DCF) with excitation around 488 nm (Kaur et al. 2016). In the present study, a 50 mM H_2_DCFDA stock solution was prepared in dimethyl sulfoxide (DMSO, purity 99.7%; Sigma-Aldrich, St. Louis, MO, USA) and stored at − 20 °C until use. After the exposure, fronds were immersed in 200 µL working solution of H_2_DCFDA (50 µM) prepared in the culture medium. After 1 h of probe loading, the fronds were rinsed with clean medium and transferred to a black 96-well microplate (Corning Incorporated, Costar^®^, NY, USA). The fluorescence of the fronds was immediately measured using a VICTOR^3^ fluorescent plate reader, 1400 Multilabel Counter (Perkin Elmer, Boston, MA, USA) with excitation/emission wavelength of 485/538 nm. The background fluorescence of the medium-probe mix (without the presence of fronds) was also measured and subtracted from the total fluorescent counts of the samples.

### Lipid peroxidation assay

Malondialdehyde (MDA), a marker of lipid peroxidation (LPO), was assessed by the thiobarbituric acid reactive substances (TBARS) method as described by Zezulka et al. (2013), with minor modifications (Xie et al. 2018). In brief, 5 mg of fronds was homogenized in 1 mL of 0.25% (w/v) 2-thiobarbituric acid (TBA, Sigma-Aldrich) in 10% trichloroacetic acid (TCA, Sigma-Aldrich) and incubated at 95 °C for 30 min. After incubation, the fronds were cooled in an ice bath for 10 min. and centrifuged at 10,000*g* for 10 min (< 4 °C). The absorbance of the supernatant was measured at 532 nm and corrected for non-specific turbidity by subtracting the absorbance at 600 nm. Absorbance at 440 nm was also used as a baseline to avoid interferences conferred by carbohydrates (Hodges et al. 1999). A background control containing 0.25% TBA in 10% TCA was also analysed and subtracted from the total absorbance in the samples. The MDA level was normalised to frond weight and presented as µmol g^−1^ using an extinction coefficient of 155 nmol^−1^ cm^−1^ (Hashem 2013).

### Mitochondrial membrane potential assay

The mitochondrial membrane potential (MMP), an indicator of oxidative phosphorylation (OXPHOS) status, was measured using tetramethyl rhodamine methyl ester (TMRM, Molecular Probes Inc). As a potentiometric dye, TMRM is widely used to evaluate the MMP in living cells and can be also taken up efficiently by sufficiently energized young plant tissues such as *Arabidopsis* roots (Teardo et al. 2015). In brief, 5 mM TMRM stock solution was prepared in DMSO and stored at − 20 °C until use. After the UVR exposure, fronds were incubated in 200 µL of TMRM (500 nM) working solution prepared in the culture medium (room temperature for 1 h) in darkness, then washed with the culture medium to remove excessive probe. Rinsed fronds were transferred to a 96-well black microplate (Corning) and the fluorescence recorded using the VICTOR^3^ plate reader with excitation/emission wavelength of 530/590 nm.

### Photosynthetic pigment content

Photosynthetic pigment content was determined spectrophotometrically as previously described by Wellburn (1994) with minor modifications. Approximately 25 mg of fresh fronds was submerged in 1.5 mL DMSO (100%), incubated at 60 °C for 1 h with sonication to disrupt the tissues. The resulting supernatant was transferred to a disposable spectrophotometer cuvette (Brand semi-micro, Wertheim, Germany) for determination of absorbances at wavelength of 649, 665 and 480 nm using a UV/Vis spectrophotometer Lambda 40 (PerkinElmer). The contents of chlorophyll *a* and *b*, and total carotenoids were calculated in μg mL^−1^using the following equations:

1 $${\text{Chorophyll }}a \left( {{\text{Chl}} a} \right) = 12.19 \times A_{665} - 3.45 \times A_{649}$$

2 $${\text{Chorophyll }}b \left( {{\text{Chl }}b} \right) = 21.99 \times A_{649} - 5.32 \times A_{665}$$

3 $${\text{Total carotenoids }}\left( {{\text{Car}}} \right) = \left( {1000 \times A_{480} - 2.14 \times Ca - 70.16 \times Cb} \right)/220$$

### Chlorophyll *a* fluorescence

The photosynthetic capacity of *L. minor* was determined as Pulse-Amplitude-Modulated (PAM) chlorophyll fluorescence kinetics using a DIVING PAM (Walz, Effeltrich, Germany). Plants were first maintained in the dark for 30 min prior to allow complete oxidation of the PSII reaction centre and initial fluorescence (*F*_o_) was then measured under weak illumination (1 μmol m^−2^ s^−1^). The measurement of maximal fluorescence (*F*_m_) was obtained by applying a saturating light pulse (5000 μmol m^−2^ s^−1^, 0.8 s), whereas steady-state terminal fluorescence (*F*_t_) and maximal fluorescence yield of the illuminated sample (*F*_m_′) were determined after 10 min of continuous illumination (PAR = 80 μmol m^−2^ s^−1^) at the equilibrium state. All fluorescence yields were used to calculate the maximal PSII efficiency (*F*_v_/*F*_m_), the effective PSII efficiency (Φ_PSII_), the coefficients of photochemical (qP) and non-photochemical quenching (NPQ) (Table 2).

**Table 2:** Fluorescence parameters calculated from pulse-amplitude modulation (PAM) fluorometry measurements in *Lemna minor* exposed to UVB radiation for 7 days

Parameter	Description	Equation	References
*F*_o_′	Effective minimal fluorescence	*F*_o_' = *F*_o_/(*F*_v_/*F*_m_ + *F*_o_/*F*_m_')	Oxborough and Baker (1997)
*F*_v_/*F*_m_	Maximal photosystem II efficiency	(*F*_m_ − *F*_o_)/*F*_m_	Maxwell and Johnson (2000)
Φ_PSII_	Effective photosystem II efficiency	(*F*_m_′ − *F*_t_)/*F*_m_′	Maxwell and Johnson (2000)
qP	Photochemical quenching	(*F*_m_′ − *F*_t_)/ (*F*_m_ − *F*_o_′)	Maxwell and Johnson (2000)
NPQ	Non-photochemical quenching	(*F*_m_ − *F*_m_′)/*F*_m_′	Maxwell and Johnson (2000)

### Transcriptional analysis

After 7 days’ exposure to UVR, six fronds from each treatment group except for 4 W m^−2^ that were all dead and discoloured were snap-frozen in liquid nitrogen and stored at − 80 °C until use. Total RNA was extracted using the Quick-RNA Plant RNA MiniPrep kit in combination with on-column DNase I treatment (Zymo Research Corp, Irvine, CA, USA), according to the manufacturer’s protocols. The RNA purity and integrity were assessed using a Nanodrop ND-1000 (Nanodrop Technologies, Wilmington, DE, USA) and a Bioanalyzer (Agilent Technologies, Santa Clara, CA, USA), respectively.

The quantitative real-time reverse transcription polymerase chain reaction (qPCR) assay was performed as previously described (Song et al. 2016). In brief, cDNA was reversely transcribed from 100 ng total RNA using qScript™ cDNA SuperMix (Quanta BioSciences, Gaithersburg, MD, USA). Primers used for qPCR were designed using the Primer 3 v0.4.0 (https://bioinfo.ut.ee/primer3-0.4.0) and purchased from Invitrogen (Carlsbad, CA, USA) (Suppl. Table S1). For amplification, 1 µg of cDNA template, 15 μL of PerfeCTa SYBR Green FastMix^®^ (Quanta BioSciences) and 400 nM of forward/reverse primer were added to a 20-μL reaction mixture. Each sample was analysed in duplicate as technical replicates. A standard curve was generated using pooled cDNA standards (0.25–4 ng). A no-reverse-transcriptase control (NRT) and a no-template control (NTC) were included in the qPCR amplification as quality controls. The qPCR analysis was conducted using a CFX384 Real-Time PCR Detection System (Bio-Rad Laboratories, Philadelphia, PA, USA). Expression of 16 target genes considered relevant to potential toxicity pathways in response to elevated UVR in plants was assessed, and the Ct values were determined for four biological replicates (each containing two technical replicates). The relative expression was calculated using the Pfaffl method (Pfaffl 2001). Gene expression data for the target genes was normalized to the geometric mean expression of three reference genes: glyceraldehyde 3-phosphate dehydrogenase (*GADPH*), 40 s ribosomal protein S18 (*RPS18*) and elongation factor 1-α (*EF1α*) (Shi et al. 2016; Tang et al. 2017). The normalized expression of each target gene was further normalized to the mean expression of the non-UV control samples.

### Presentation of results, statistical analyses and graphical methods

The results (growth parameters, photosynthetic pigment content, chlorophyll *a* fluorescence) are presented as relative values with the response of the control defined as 0% and the maximal response defined as 100%. The CPD levels, ROS levels, degree of lipid peroxidation and mitochondrial membrane potential were presented as fold change compared to the non-UV control. The raw fluorescent counts were normalized by the weight of the fronds for the ROS and MMP assays. The results for each endpoint were presented as the mean of four replicates with standard error (mean ± SE). The expression of biomarker genes was displayed by a boxplots depicting the median, quartiles, minimum and maximum value. All statistical analyses and figures were made by GraphPad Prism version 6 (GraphPad Software, San Diego, CA, USA). Data were assessed for normality and equal variance by Shapiro–Wilk and Levene’s tests. Statistical analysis was performed using both the non-UV control, non-UV-CT (no UVA and no UVB) and UVA control, UVA-CT (low UVA and low UVB irradiance) to identify potential contribution of low UVA irradiance to the UVB exposure groups. Significance was determined using a one-way analysis of variance (one-way ANOVA) followed by Dunnett’s multiple comparison test using a significance level of *P* ≤ 0.05 to characterize the No Observed Effect Irradiance (NOEI) and Lowest Observed Effect Irradiance (LOEI). Then 50% Effect Irradiance (EI_50_) was derived from the sigmoidal irradiance-response curves (IRC) for parameters having fully characterized IRCs. Two-way ANOVA was also employed to analyse changes of exposure conditions including irradiances, pH and temperature. A principal component analysis (PCA) was applied to identify potential correlations between the responses of adverse effects, functional endpoints and targets genes using the XLSTAT2018 program (Addinsoft, Paris, France). The same software was used to calculate Pearson’s correlation coefficients between different endpoints (*P* ≤ 0.05).

## Results

After exposure for 7 days, the results show that the UVA control (UVA-CT) did not cause any significant alterations in the measured biological endpoints and the expression of biomarker genes compared to the non-UV control (non-UV-CT). As a consequence, UVA-CTs were considered to represent a low UVB group having the same UVA irradiance as all other exposure groups and included in the irradiance-response curve (IRC) and statistical analyses. UVB caused both irradiance-dependent and target-specific effects in *L. minor* compared to the non-UV-CT (and UVA-CT), where detailed presentation is given in the subsequent subsections.

### Growth responses

**Fig. 1:**
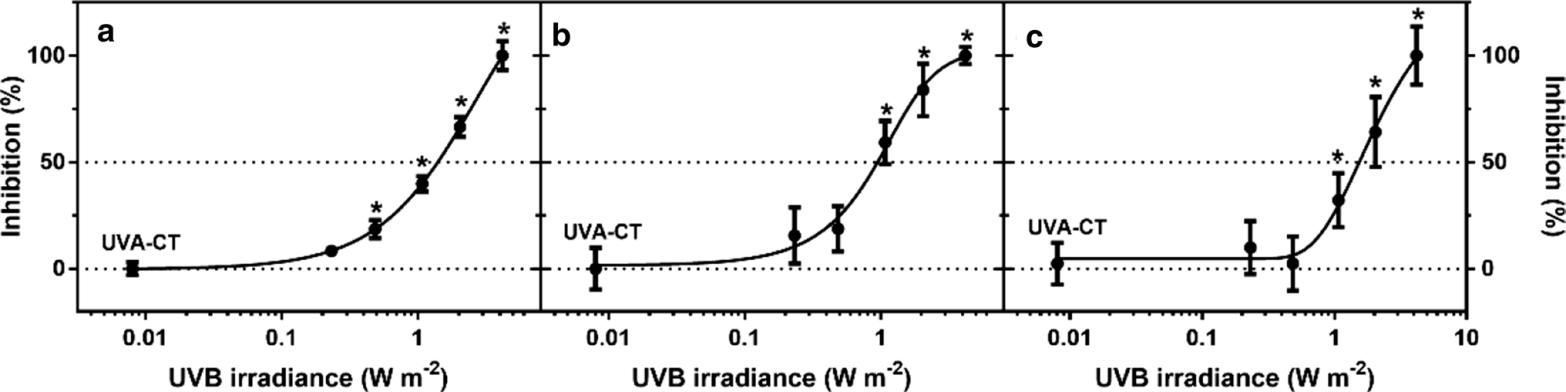
Growth inhibition measured as percent (%) reduction in **a** frond number, **b** frond area and **c** dry weight relative to the non-UV control in *Lemna minor* exposed to different irradiances of UVB radiation for 7 days (mean of 4 replicates ± SE). The dotted line indicates the non-UV control and 50% effective irradiance (IE_50_) levels, and the solid line shows the fitted non-linear regression curve, whereas the asterisks (*) indicate significant differences compared to the UVA control (UVA-CT) (*P* ≤ 0.05)

Exposure to UVB caused growth inhibition, measured as an irradiance-dependent reduction in frond number (FN), frond area (FA) and dry weight (DW) compared to the non-UV control (Fig. 1). The LOEIs for UVB were observed at 0.5–1 W m^−2^, the largest growth inhibition occurred at 4.2 W m^−2^, whereas the EI_50_ for the growth parameters ranged between 0.98 and 2.6 W m^−2^, respectively (Table 3). After 7 days of exposure, the frond growth (reproduction) rate in the non-UV control and UVA control were 0.325 ± 0.004 and 0.332 ± 0.005 day^−1^, respectively, which were above the validity criterion of the OECD guidelines (OECD 2006) (*R* = 0.275 day^−1^).

**Table 3:** No observed effect irradiance (NOEI), no observed effect dose (NOED), lowest observed effect irradiance (LOEI), lowest observed effect dose (LOED), 50% effect irradiance (EI_50_) and 50% effect dose (ED_50_; mean of 4 replicates ± SE) and regression coefficient (*R*^2^) of selected endpoints in *Lemna minor* after 7-day exposure to different irradiances of UVB radiation

Endpoints	NOEI (W m^−2^)	NOED (KJ m^−2^)	LOEI (W m^−2^)	LOED (KJ m^−2^)	EI_50_ (W m^−2^)	ED_50_ (KJ m^−2^)	*R*^2^
FN	0.23	140.8	0.48	293.9	1.26 ± 0.14	762 ± 84	0.989
FA	0.48	293.9	1.08	650.1	0.89 ± 0.12	538 ± 72	0.936
DW	0.48	293.9	1.08	650.1	1.51 ± 0.24	907 ± 145	0.903
ROS	0.23	140.8	0.48	293.9	N/A	N/A	0.944
LPO	0.23	140.8	0.48	293.9	N/A	N/A	0.939
CPD	0.008	4.8	0.23	140.8	N/A	N/A	0.962
MMP	0.48	293.9	1.08	650.1	N/A	N/A	0.975
*F*_v_/*F*_m_	0.23	140.8	0.48	293.9	2.01 ± 0.31	1216 ± 187	0.858
Φ_PSII_	0.008	4.8	0.23	140.8	0.89 ± 0.12	175 ± 72	0.968
qP	0.008	4.8	0.23	140.8	0.68 ± 0.09	411 ± 54	0.958
NPQ	0.008	4.8	0.23	140.8	0.22 ± 0.05	133 ± 30	0.975
Chl *a*	0.48	293.9	1.08	650.1	1.29 ± 0.24	780 ± 145	0.906
Chl *b*	0.48	293.9	1.08	650.1	1.06 ± 0.13	641 ± 78	0.947
Car	0.23	140.8	0.48	293.9	1.79 ± 0.28	1082 ± 169	0.851

*FN* frond number, *FA* frond area, *DW* dry weight, *ROS* reactive oxygen species, *LPO* lipid peroxidation, *CPD* cyclobutane pyrimidine dimers, *MMP* mitochondrial membrane potential, *F*_*v*_*/F*_*m*_ maximum quantum yield of photosystem II (PSII), *Φ*_*PSII*_ effective PSII efficiency, *qP* photochemical quenching, *NPQ* non-photochemical quenching, *Chl a* chlorophyll a, *Chl b* chlorophyll b, *Car* total carotenoids, *N/A* not applicable/achieved

### Oxidative stress and lipid damage

**Fig. 2:**
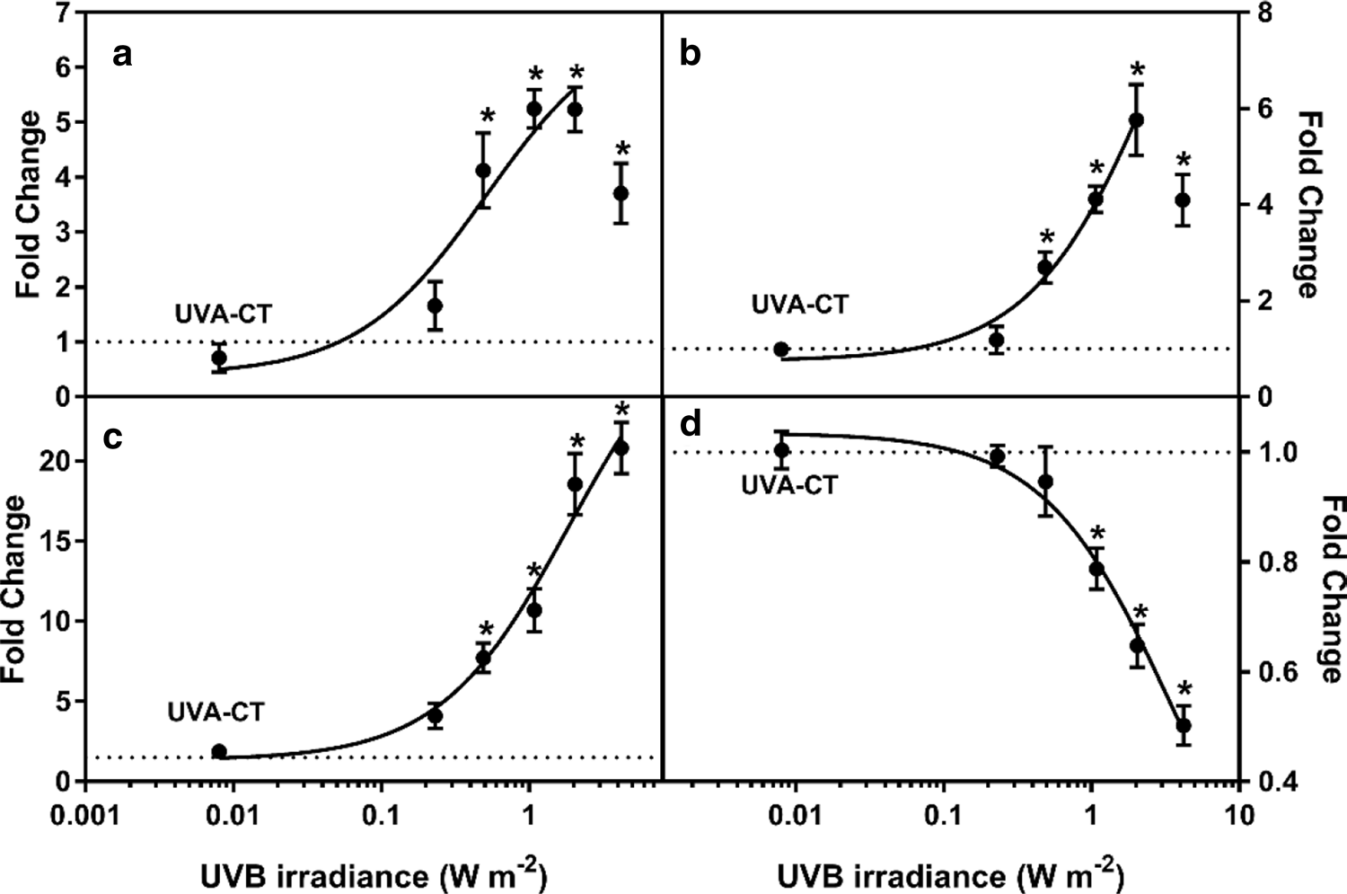
UV-induced changes in **a** reactive oxygen species (ROS) formation, **b** lipid peroxidation (LPO), **c** cyclobutane pyrimidine dimer (CPD) formation and **d** mitochondrial inner membrane potential (MMP) relative to the non-UV control in *Lemna minor* after 7 days’ exposure to different irradiances of UVB (mean of 4 replicates ± SE). The dotted line indicates the non-UV control. The results are presented as fold change compared to the blank control and the solid line shows the fitted non-linear regression curve, whereas the asterisks (*) indicate significant differences compared to the UVA control (UVA-CT) (*P* ≤ 0.05). Mortality at the irradiances above 2 W m^−2^ precluded reliable analysis of ROS and LPO, but the points were still included for illustration purposes

UVB caused an irradiance-dependent induction of ROS formation and LPO (measured as MDA content) in *L. minor* after 7 days of exposure (Fig. 2a, b). UVB significantly increased both ROS and MDA formation in *L. minor* from 0.48 W m^−2^ (LOEI) (Table 3). Both ROS formation and MDA content reached a plateau at 2.0 W m^−2^ UVB and then decreased at 4.2 W m^−2^.

### Cyclobutane pyrimidine dimer formation and mitochondrial membrane potential

Increased UVB irradiance induced an irradiance-dependent induction of CPD. Significant induction of CPD content in *L. minor* was observed from 0.23 W m^−2^ (LOEI) (Table 3). The MMP in *L. minor* decreased in an irradiance-dependent manner after 7-day exposure to UVB (Fig. 2d), and the LOEI for MMP was determined to be 1.1 W m^−2^ (Table 3).

### Photosynthesis efficiency

**Fig. 3:**
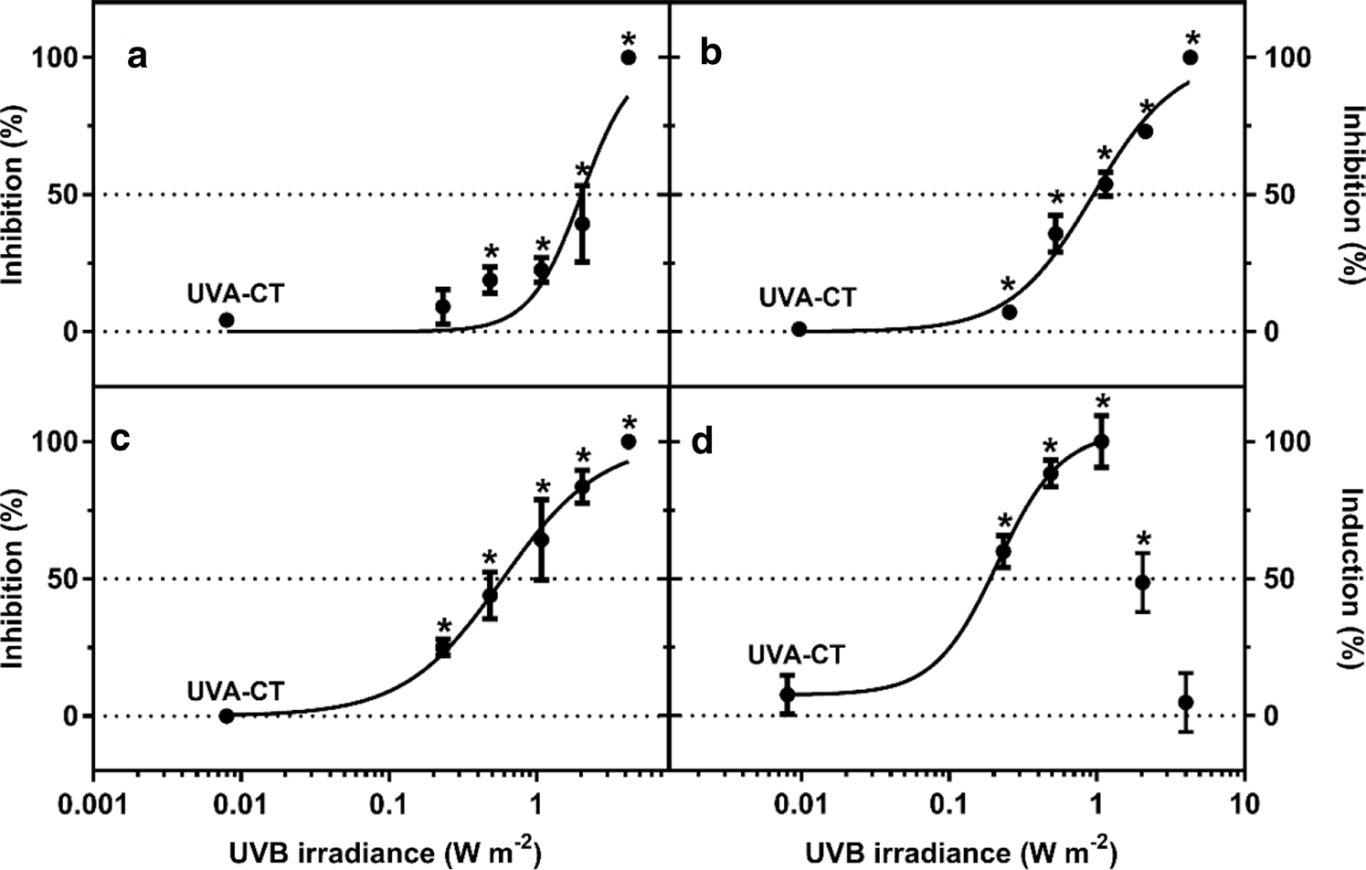
Inhibition of photosystem II (PSII) performance as **a** maximal PSII efficiency (*F*_v_/*F*_m_), **b** effective PSII efficiency (Φ_PSII_), **c** photochemical quenching (qP) and **d** non-photochemical quenching (NPQ) in *Lemna minor* after 7 days’ exposure to different irradiances of UVB radiation (mean of 4 replicates ± SE). The dotted line indicates the non-UV control and 50% effective irradiance (IE_50_) levels, and the solid line shows the fitted non-linear regression curve, whereas the asterisks (*) indicate significant differences compared to the UVA control (UVA-CT) (*P* ≤ 0.05). Mortality at the irradiances above 2 W m^−2^ precluded reliable analysis of NPQ, but the points were still included for illustration purposes

The exposure of *L. minor* to UVB resulted in an irradiance-dependent inhibition of PSII efficiency (Fig. 3). The 7-day EI_50_ of *F*_v_/*F*_m_, Φ_PSII_ and qP ranged between 0.68 and 2.0 W m^−2^ (Table 3), whereas LOEI of *F*_v_/*F*_m_, Φ_PSII_ and qP was determined at 0.23, 0.89 and 0.68 W m^−2^, respectively. Additionally, a significant enhancement of NPQ yield was observed in *L. minor* after exposure to 0.23 W m^−2^ UVB, followed by a reduction at 2.0 W m^−2^. Complete growth inhibition precluded the analysis of NPQ at the highest irradiance (4.2 W m^−2^).

### Content of pigments

**Fig. 4:**
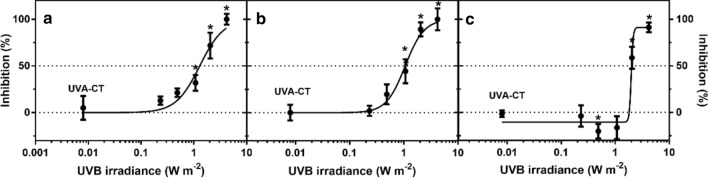
Reduction of the content of **a** chlorophyll *a*, **b** chlorophyll *b* and **c** total carotenoids in *Lemna minor* after 7 days’ exposure to different irradiances of UVB radiation (mean of 4 replicates ± SE). The dotted line indicates the non-UV control and 50% effective irradiance (IE_50_), and the solid line shows the fitted non-linear regression curve, whereas the asterisks (*) indicate significant differences compared to the UVA control (UVA-CT) (*P* ≤ 0.05)

Irradiance-dependent decrease in chlorophyll *a* (Chl *a*) and chlorophyll *b* (Chl *b*) was observed in *L. minor* after 7-day exposure to UVB (Fig. 4). A significant reduction in pigment content was noticed at 1.1 W m^−2^ (LOEI) for both Chl *a* and Chl *b*, with EC_50_ at 1.3 and 1.1 W m^−2^ (Table 3), respectively. Under UVB exposure, the content of total carotenoids (Car) was significantly inhibited at 2.0 and 4.2 W m^−2^, but was enhanced at 0.48 W m^−2^. The EI_50_ for the reduction of carotenoid content was estimated to be 1.8 W m^−2^.

### Transcriptional responses

**Fig. 5:**
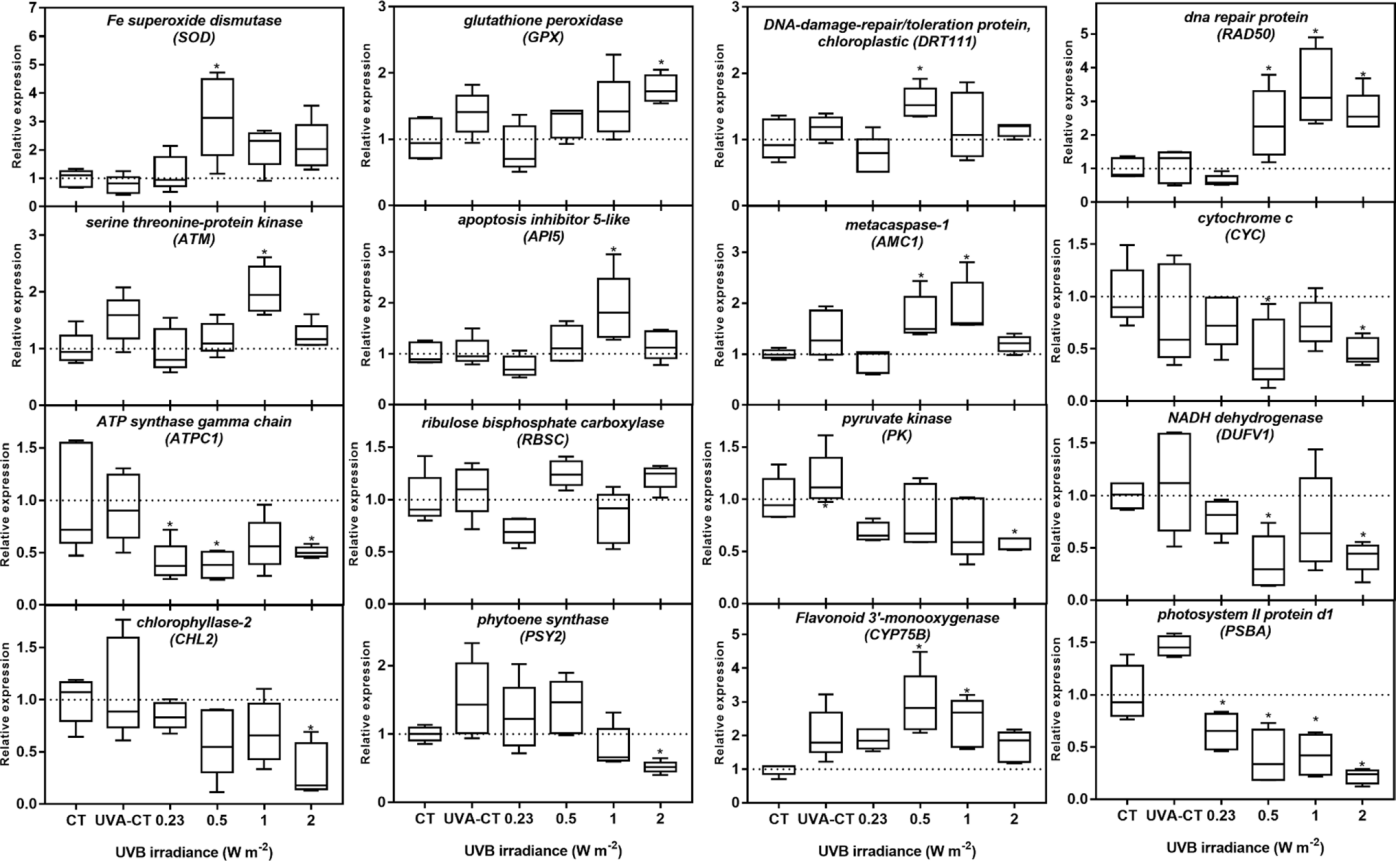
Relative transcript levels in *Lemna minor* after 7 days’ exposure to different irradiances of UVB radiation. The data are derived from quantitative real-time reverse transcription polymerase chain reaction analyses (qRT-PCR, white box, *n* = 4 biological replicates, each contains 2 technical replicates). The transcript levels were normalized against the housekeeping gene glyceraldehyde 3-phosphate dehydrogenase (*Gadph*), 40 s ribosomal protein S18 (*RPS18*), and elongation factor 1-α (*EF1α*) and are shown relative to the non-UV control (shown as dotted line). The asterisks (*) indicate significant differences compared to the CT (non-UV control) (*P* ≤ 0.05) and UVA-CT refers to the UVA control. Boxes represent the median and the 25th and 75th percentiles and the whiskers represent the minimum and maximum value

The genes encoding the antioxidant enzymes superoxide dismutase (*SOD*) and glutathione peroxidase (*GPX*), the DNA damage repair-related genes DNA damage-repair/toleration protein (*DRT111*) and DNA repair protein rad50 (*RAD50*), the DNA damage sensor serine threonine-protein kinase (*ATM*), the cell death-related genes apoptosis inhibitor 5-like (*API5)* and metacaspase-1 (*AMC1*) as well as a gene involved in production of UV-protecting flavonoids, flavonoid 3′-monoxygenase (*CYP75B*), were all significantly up-regulated at a UVB irradiance of 0.5–2.0 W m^−2^ compared to the non-UV control after 7 days’ exposure (Fig. 5). Genes encoding the chlorophyll degradation enzyme chlorophyllase-2 (*CHL2*), the carotenoid biosynthesis enzyme phytoene synthase (*PSY2*), the PSII protein D1 (*PSBA*), the chloroplast ATP synthase gamma chain (*ATPC1*), the glycolysis enzyme pyruvate kinase (*PK*), the OXPHOS/mitochondrial electron transport genes NADH dehydrogenase (*NDUFV1*) and cytochrome c (*CYC*) were all significantly down-regulated at one or more irradiances. The gene encoding the small subunit of the CO_2_ fixation protein ribulose bisphosphate carboxylase (*RBSC*) was not differentially expressed at any UVB irradiance. Irradicne and dose thresholds for gene responses (i.e. NOEI, NOED, LOEI and LOED) are provided in Suppl. Table S4.

### Principal component analysis (PCA)

**Fig. 6:**
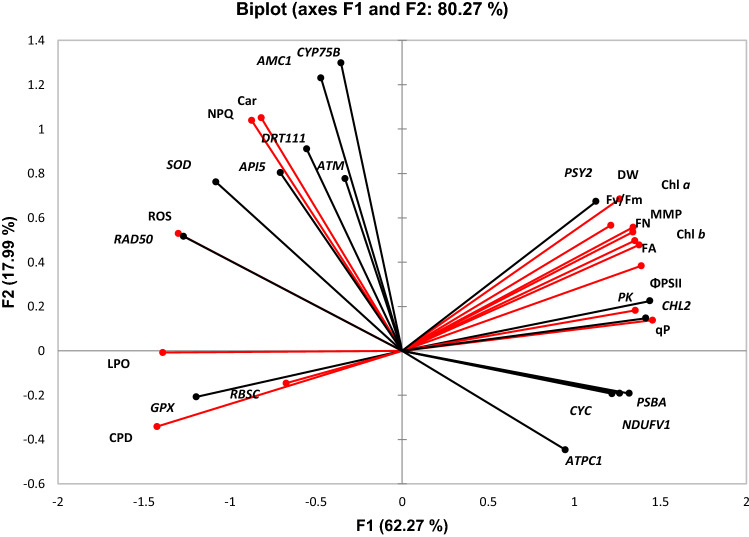
Principal component analysis (PCA) of adverse effects and mechanistic responses together with the expression of related genes in *Lemna mino*r exposed to UVB radiation for 7 days (*n* = 4 replicates). The normal text with red line indicates the endpoints: *FN* frond number, *FA* frond area, *DW* dry weight, *ROS* reactive oxygen species, *LPO* lipid peroxidation, *CPD* cyclobutane pyrimidine dimer, *MMP* mitochondrial membrane potential, *F*_*v*_*/F*_*m*_ maximum quantum yield of photosystem II (PSII), *Φ*_*PSII*_ effective PSII efficiency, *qP* photochemical quenching, *NPQ* non-photochemical quenching, *Chl a* chlorophyll *a*, *Chl b* chlorophyll *b*, *Car* total carotenoids. Italic texts with black line indicate the genes: *SOD* superoxide dismutase, *GPX* glutathione peroxidase, *DRT111* DNA damage-repair/toleration protein, *ATM* serine threonine-protein kinase, *Rad50* DNA repair protein rad50, *API5* apoptosis inhibitor 5-like, *AMC1* metacaspase-1, *CYP75B* flavonoid 3′-monoxygenase, *CHL2* chlorophyllase-2, *PSY2* phytoene synthase, *PSBA* photosystem II protein D1, *ATPC*1 ATP synthase gamma chain, *PK* pyruvate kinase, *NDUFV1* NADH dehydrogenase, *CYC* cytochrome c, *RBSC* ribulose bisphosphate carboxylase

The two principal components (PC1 and PC2) of the PCA explained as much as 80.3% of the total variance (Fig. 6). The major variance (PC1 = 62.3%) was explained by growth parameters, modulation of PSII performance, reduction in chlorophyll content and increase in oxidative stress and DNA damage. The PC2 explained 18% of total variance, where NPQ and carotenoids together with the flavonoid synthesis-related gene *CYP75B* contributed mostly to the variance. A Pearson correlation analysis (Suppl. Table S2) revealed that inhibition of growth (in terms of front number FN, front area FA, and DW) was positively correlated with the reduction in MMP, PSII performance (*F*_v_/*F*_m_, Φ_PSII_ and qP), chlorophyll content (Chl *a* and Chl *b*) and down-regulation of the genes *PK*, *CHL2* and *PSY2*, but negatively correlated with the increase of CPD, LPO and up-regulation of the gene *GPX*.

## Discussion

Although the ambient UVB levels are normally not harmful to plants, elevated UVB irradiance due to damage of the ozone layer and/or the presence of artificial UV sources may have detrimental effects on aquatic primary producers if they are not properly acclimated to such exposures (Jansen 2012). In this respect, UVB can reduce primary productivity and influence competitive interactions either in ecosystems or during cultivation (Williamson et al. 2019). The present study aimed at providing mechanistic insights into how continuous and elevated UVB affects different physiological processes in the model macrophyte duckweed. All the endpoints and expression of biomarker genes were measured as an initial effort to characterize the combination of acute and compensatory responses occurring after a chronic 7-day-exposure. The results clearly showed irradiance-dependent and target-specific changes consistent with current knowledge of UVB effects in primary producers, albeit some inconsistencies were also identified.

### Exposure conditions

The UVB irradiances (from 0.23 to 2.0 W m^−2^) used in the current study covered the natural UVB irradiance ranges in Northern Europe (Suppl. Fig. S4). The highest UVB irradiance, representing twice the maximal natural UVB levels (as estimated by the NCAR TUV calculator; NCAR 2018), was included to ensure capturing full irradiance-response curves (IRCs) for the toxicity endpoints determined. Compared to the natural environment in northern Europe during summer, the total UVB dose of the lowest irradiance (0.23 W m^−2^) used in the present study was less than the total daily integrated dose of UVB, whereas exposure to the highest irradiance (4.2 W m^−2^) was 14-fold higher than the normal daily integrated dose (Johnsen 2020; Suppl. Table S3). Standardized laboratory testing protocols and other *Lemna* studies have recommended using PAR levels around 80–100 µmol m^−2^ s^−1^ (Moody and Miller 2005), which yield higher UVB:PAR ratios than that occurring under natural conditions. Such conditions are also expected to occur in aquaponics and hydroponics receiving artificial lightening in situations where UV-irradiation is used to reduce or eliminate UVB-sensitive phytopathogens by disrupting DNA replication (Van Os et al. 2004; Mori and Smith 2019). Although this may render plants such as *L. minor* more sensitive to UVB than exposures under natural solar UVB:PAR ratios (Krizek 2004), characterising the toxicity pathways leading to growth effects are of significant interest particularly for cultivation under artificial light and UVR conditions and may subsequently assist in identifying how UVB can act in combination with other environmental or anthropogenic stressors to cause adverse effects (Teramura 1986). Non-UVB parameters including PAR, UVA irradiance and temperature were kept constant throughout the study (Suppl. Fig. S2 and Suppl. Fig. S3) to minimize the influence from other factors than UVB.

However, a slight increase in pH (from 6.52 to 7.11) was observed, potentially due to the root exudate of inorganic ions such as HCO_3_^−^ and OH^−^ into the growth media to maintain electrical neutrality (Dakora and Phillips 2002). This pH shift (≤ 10%) was considered small and changes of this magnitude have not been demonstrated to alter the growth of *L. minor* in earlier studies (McLay 1976). Nevertheless, the present study suggests implementing media change every 2–4 days to minimize any pH shift during chronic exposure studies.

### Growth

UVB irradiance significantly inhibited the asexual reproduction (front number FN), morphology (front area FA) and biomass (DW) of *L. minor* in an irradiance-dependent manner (Fig. 1). Among those three parameters, FA was the most sensitive endpoint (EI_50_ = 0.89 W m^−2^), closely followed by FN (EI_50_ = 1.26 W m^−2^) and DW (EI_50_ = 1.51 W m^−2^) (Table 3). UVB-induced reduction in leaf expansion has previously been associated with altered transcription of hormone-metabolism genes, and thus reduced content of the growth-stimulating plant hormones indoleacetic acid (IAA) and gibberellin (GA), resulting in reduced cell proliferation and expansion (Roro et al. 2017). UVB radiation is also known to act through the UVB photoreceptor UVR8, which has been associated with changes to the circadian clock, stomatal closure, leaf development, osmotic stress, etc. (Ulm and Jenkins 2015).

### ROS formation and lipid peroxidation

Enhanced endogenous ROS formation is one of the initial reactions to UVB exposure (Hideg et al. 2013). The present results suggest that cellular ROS production was one of the most sensitive responses in *L. minor* exposed to UVB, with significant induction already at 0.48 W m^−2^ (Table 3, Fig. 2). Increase in ROS production is known to disrupt cellular homeostasis and cause oxidative stress in plants, which may lead to oxidative damage to the membrane lipids, nucleic acids and proteins (Mittler 2002). When exposed to UVB, both the mitochondria and the chloroplasts are key producers of endogenous ROS in plants (Nawkar et al. 2013). Under normal circumstances, the production and the elimination of ROS in plants are in a dynamic equilibrium with antioxidants regulating the plant’s tolerance to oxidative damage (Foyer et al. 1994). In the present study, prolonged UVB radiation caused up-regulation of the antioxidant-related gene *SOD* already at 0.48 W m^−2^, which suggests that superoxide was the major ROS in *L. minor* at low UVB irradiances, as the enzyme SOD catalyses the dismutation of superoxide into oxygen and hydrogen peroxide (Lubos et al. 2011). An increase in transcription of *GPX*, whose main role is the enzymatic dismutation of hydrogen peroxide into oxygen and water (Lubos et al. 2011), occurred at 2.0 W m^−2^ and higher irradiances and suggested substantial accumulation of hydrogen peroxide at higher UVB levels. The upregulation of the two genes (*SOD* and *GPX*) is in agreement with the study in *Glycine max*, where UVB-induced enhancement of superoxide and hydrogen peroxide was observed at 0.4 W m^−2^ (Prasad et al. 2005). When endogenous ROS production exceeds the antioxidant capacity, ROS can cause damage to the lipids as demonstrated by the increased LPO from 0.48 W m^−2^ in the current study. Similar enhanced ROS-induced LPO in chloroplast membranes has also been demonstrated in rice after exposure to 22 W m^−2^ UVB for 7 days (Lidon and Ramalho 2011).

### DNA damage

DNA is one of the most well-studied targets of UVB-induced damage in plants. UVB radiation can generate cytotoxic DNA lesions such as the formation of CPDs and 6–4 photoproducts (6-4PPs) (Jansen et al. 1998), where CPDs account for about 90% of all UVB-induced pyrimidine dimers in plants (Dany et al. 2001). In the present study, UVB-induced DNA lesions were evidenced by a combination of increased CPD formation and up-regulation of DNA damage response genes (Fig. 2). The results clearly showed significant CPD generation in the fronds after exposure to 0.48 W m^−2^ and higher UVB irradiation. Increased CPD has also been observed in *Oryza sativa* and *Arabidopsis thaliana* after exposure to 1 W m^−2^ UVB (Teranishi et al. 2004), thus suggesting that *L. minor* may be more sensitive to UVB than some terrestrial plants. Some of this sensitivity can be attributed to the low UVA irradiance used in the present study, as UVA radiation can initiate photoreactivation to repair UVB-induced CPD in plants (Jansen et al. 1998). The lack of differences in CPD between the non-UV control and the UVA control indicated that the UVA irradiance used was too low to trigger photooxidation as no difference in baseline CPD was determined. In addition to CPDs generation, the DNA damage repair and regulatory genes, *DRT111*, *RAD50* and *ATM*, were also up-regulated at 0.48 W m^−2^ and/or 1.1 W m^−2^ (Table 3), suggesting that UVB at high levels might also induce DNA damage and repair processes associated with double-strand breaks (DSB) in *L. minor*. DNA double-strand break occurring under UVB exposure in plants can be caused by either increased oxidative stress, or direct induction of CPD formation (Rastogi et al. 2010). Studies with *Arabidopsis* have shown that DSB can activate the *ATM* (ATM serine/threonine kinase) signaling to trigger the activation of *SOG1*, the central regulator of the DNA damage responses (DDRs) and plant-specific transcription factor, that consequently results in slower cell division and programmed cell death (PCD) (Furukawa et al. 2010). Such DNA damage-induced disruption of DNA replication and transcription has been documented to affect cellular function and inhibit normal growth in a number of crops including rice, spinach, cucumber, tomato, wheat and barley (Manova and Gruszka 2015).

### Programmed cell death

In the present study, up-regulation of programmed cell death-related genes such as *API5* and *AMC1* in the range from 0.5 to 1.1 W m^−2^ suggests that UVB-enhanced PCD occurred in *L. minor* (Fig. 5)*.* UVB-mediated PCD was also observed in tobacco cells after 5.6 W m^−2^ exposure for 12 h (Lytvyn et al. 2010). In general, *API5* encodes an inhibitory protein that prevents PCD in plants after growth factor deprivation, whereas the gene *AMC1* encodes metacaspases-1 that acts as a positive regulator enzyme involved in oxidative stress-induced cell death (Arambage et al. 2009). The increased expression of *API5* and *AMC* was only significantly correlated to the upregulation of the *ATM* and *RAD50* genes*,* suggesting that PCD in *L. minor* could potentially be induced by DNA strand breaks.

### Oxidative phosphorylation

Oxidative phosphorylation (OXPHOS) is a key metabolic pathway involving electron transfer by electron carriers and protein complex-associated redox-reactions in the mitochondrial electron transport chain (mETC) that ultimately lead to the synthesis of ATP. UVB reduced MMP, a measure of the transmembrane potential, at intermediate irradiances (1 W m^−2^ and higher) that suggests a reduction in OXPHOS after 7 days of exposure*.* In the macroalga *Ecklonia cava*, UVB has been proposed to reduce MMP at irradiance as low as 0.1 W m^−2^ (Kim et al. 2014). Additionally, down-regulation of the mETC-relevant genes *CYC* (mitochondrial cytochrome *c*) and *NDUFV1* (NADH dehydrogenase) from 0.5 W m^−2^ also indicate a reduction in OXPHOS, as the mitochondrial cytochrome *c* and NADH dehydrogenase act as electron carriers in OXPHOS (Lodish et al. 2000; Klodmann et al. 2010). Deactivation of NADH dehydrogenase was also proposed to be a major response in the algae *Chondracanthus teedei* after exposure to 3.5 W m^−2^ UVB for 7 days (Schmidt et al. 2012). One of the major cellular effects of OXPHOS suppression is the reduction in the ATP synthesis that may limit growth in primary producers (Gruenhagen and Moreland 1971).

### Pigments

The contents of photosynthetic pigments are reliable endpoints to assess the impacts of environmental stressors on plants (Hu et al. 2013). A large part of the photosynthetic pigments is in the light-harvesting complexes (LHC) and is tightly bound to the reaction centers, thus associated with the photosynthetic capacity of plants. In the current study, enhanced UVB (≥ 0.48 W m^−2^) reduced chlorophyll content in *L. minor* fronds after 7-day exposure (Fig. 4a, b) and coheres well with similar findings in *Pisum sativum* at an irradiance of 0.27 W m^−2^ (Strid and Porra 1992). Although not assessed in detail, the study of Strid and Porra (1992) proposes that the reduction of chlorophyll in *Pisum sativum* was caused by UVB-induced photooxidation. ROS-dependent inhibition of chlorophyll biosynthesis, potentially through inhibition of magnesium-protoporphyrin IX monomethyl ester (oxidative) cyclase, offers an alternate explanation for similar observations in *Cucumis sativus* (Aarti et al. 2006). In the present study, down-regulation of the chlorophyll metabolism-related gene (*CHL2*) at high UVB irradiance (2.0 W m^−2^) suggested UVB-mediated interference with chlorophyll degradation by reducing hydrolysis of chlorophyll. Suppressed chlorophyllase activity has also been observed in *Brassica oleracea* after exposure to UVB with a total dose of 8.8 kJ m^−2^ (Kaosamphan et al. 2010). An ultimate consequence of such loss of chlorophyll may be reduction in light absorption capacity and reduction of photosynthesis (Habash et al. 1994).

Some carotenoids in plants act as inducible protective agents against oxidative damage (i.e. antioxidants) by dissipating excess energy from the photoreaction centers (Demmig-Adams 1990). The slight induction of carotenoids (Car) observed at 0.48 W m^−2^ after 7 days of UVB exposure in *L. minor* (Fig. 4) can potentially be due to enhanced antioxidant responses and is consistent with UVB-mediated increase in carotenoids in species such as *Rumex vesicarius* and *Sisymbrium erysimoides* after 6-day exposure to UVB (Salama et al. 2011). On the other hand, the reduction of carotenoids at high UVB irradiances (≥ 1 W m^−2^) (Fig. 4) may indicate impairment of this mechanism, similar to responses observed in cyanobacteria (*S. platensis*) and basil (*Ocimum basilicum*) at UVB irradiances of 0.6–4.7 W m^−2^ (Xue et al. 2007; Mosadegh et al. 2019). Since carotenoids are also important accessory light harvesting pigments, the reduction of carotenoids at higher UVB irradiances may also be due to general down-regulation of genes associated with biosynthesis of the photosynthetic pigments and reduced formation of photosystems in the chloroplasts (Jordan et al.^1994^; Marwood and Greenberg 1996). In the present study, the carotenoid synthesis-related gene *PSY2* was down-regulated at high UVR irradiance, which may partly explain the reduction of carotenoid content.

Flavonoids are well known as UV-induced pigments protecting against UV-related damage due to their UV-screening and antioxidant activity (Løvdal et al. 2010). The up-regulation of *CYP75B* (flavonoid 3′-monooxygenase) in *L. minor* at 0.48 and 1.1 W m^−2^ suggests that flavonoid production is activated to compensate for either a loss of flavonoids or an increased need for flavonoids for UVB protection as observed in *Arabidopsis thaliana* after exposure to 0.15 W m^−2^ UVB (Lois 1994).

### PSII activity

Photosystem II has been proposed to be the main site for UVB damage to photosynthesis (Teramura and Sullivan 1994). After UVB exposure for 7 days, significant inhibition of PSII performance (*F*_v_/*F*_m_, Φ_PSII_ and qP) was observed in *L. minor* (Fig. 3). The reduction in *F*_v_/*F*_m_ at 0.48 W m^−2^ and higher irradiances indicated significant photoinhibition, whereas the reduction of Φ_PSII_ and qP from 0.23 W m^−2^ demonstrated that suppression of photosynthesis also occurred at lower UVB irradiances. Moreover, down-regulation of the D1 biosynthesis gene *PSBA* from 0.23 W m^−2^ suggests that UVB interferes with normal biosynthesis/biodegradation of the D1 protein and subsequent repair of PSII. Although not assessed in the current study, UV-induced photodamage in the oxygen-evolving complex (OEC) offers additional explanations for the observed suppression of the PSII activity (Ohnishi et al. 2005).

NPQ can protect plants from excessive light energy by dissipating it into heat as a non-photochemical quenching mechanism in PSII (Müller et al. 2001). In the present study, NPQ activity increased at low irradiances (≤ 1 W m^−2^), which is consistent with observations in sessile oak *Quercus petraea* exposed to 0.15 W m^−2^ UVB for 8 h per day for 6 weeks (Szőllősi et al. 2008). The NPQ activity, which is induced by the proton gradient across the thylakoid membranes (ΔpH) (Ruban 2016), is expected to increase in situations with reduced ATP consumption in the chloroplast (Livingston et al. 2010a, b). Therefore, it can be hypothesized that UVB directly reduces the activity of the Calvin cycle, which in turn leads to a reduction in the overall ATP consumption causing a lower luminal pH. This may potentially lead to reduced ATP synthase gene expression (Fig. 5). Despite the activity of Calvin cycle was not directly analysed in the present study, UVB has been reported to specially suppress the activity of the carbon-fixation by the enzyme Rubisco in other plants such as *Brassica napus*, *Oryza sativa* and *Pisum sativum* (Kataria et al. 2014). Interestingly, UVB did not cause any significant change in the transcription of small rubisco subunit gene *RBSC* in our study, thus indicating that UVB-mediated damage of this key enzyme in the Calvin cycle is potentially due to oxidative modification and/or degradation of the large subunit of the enzyme as suggested elsewhere (Wilson et al. 1995; Kataria et al. 2014). In contrast, a reduction of NPQ was observed at high irradiances (1–4 W m^−2^) of UVB (Fig. 3d), which was found to be consistent with similar responses in cucumber and tomato after exposure to 2.4 W m^−2^ UVB (Moon et al. 2011). It seems plausible that the reduction of NPQ at high irradiances was either induced by the observed PSII-inhibition causing reduction of electron transport and thus proton transport (and ΔpH) across the thylakoid membranes (Tikhonov 2017), or increase in membrane ion permeability due to ROS-induced oxidative damage to the membranes themselves (Strid et al. 1994).

### Toxicity pathway development

**Fig. 7:**
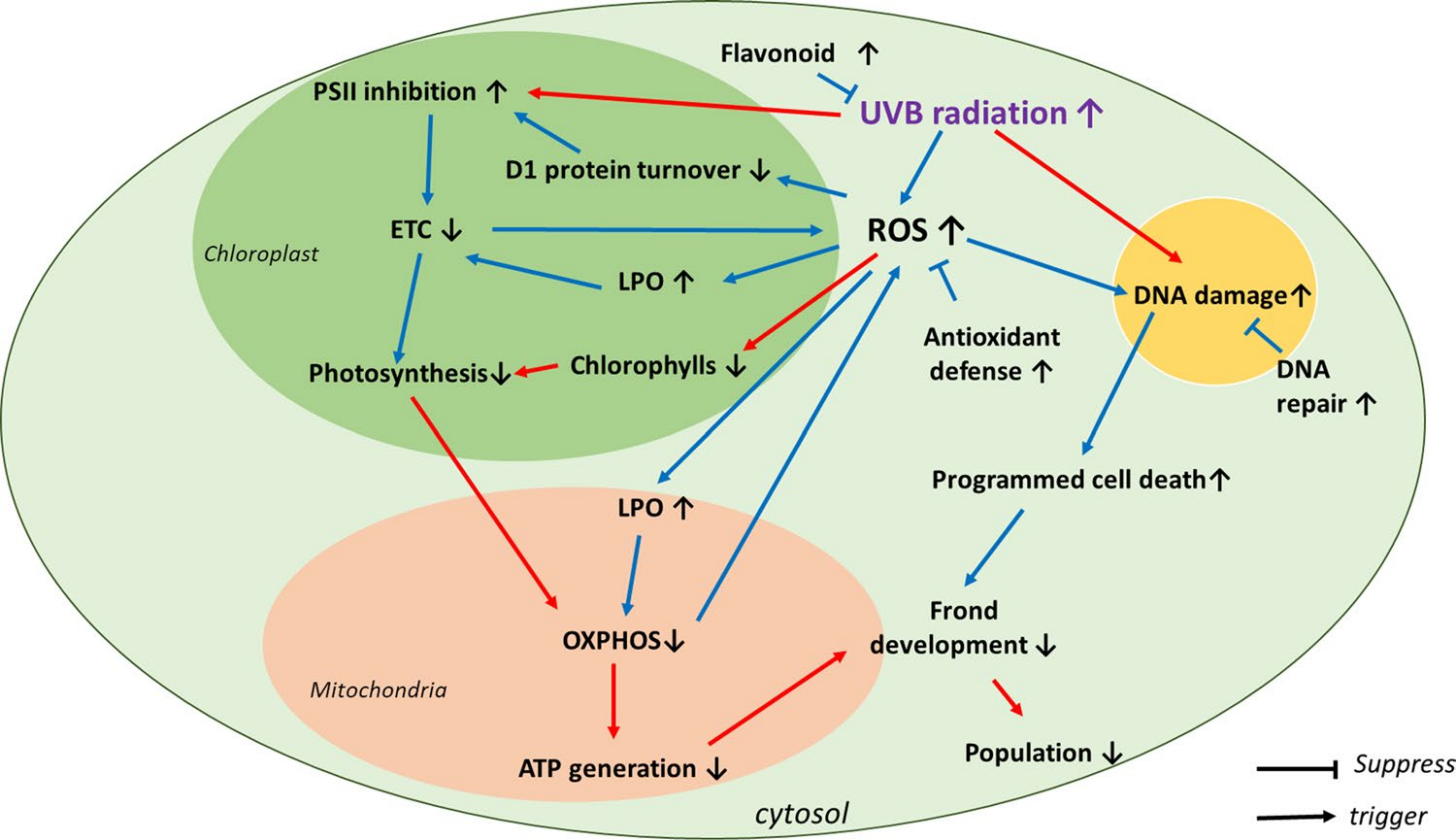
Putative toxicity pathways of UVB radiation-induced growth inhibition in the aquatic primary macrophyte *Lemna minor*. Blue arrows indicate the responses occurring at 0.23 and 0.48 W m^−2^ and the red arrows indicate the responses occurring at higher UVB irradiances (1.2–4 W m^−2^)

The present study and correlational analysis performed suggest that exposure to UVB activates irradiance-dependent toxicity pathways in *L. minor* at different biological levels of organization (Fig. 7), which may affect frond development. Induction of DNA damage and repair activity in the nucleus, programmed cell death, lipid peroxidation and interference with membrane functions associated with the ETC in both the chloroplast and the mitochondria were likely consequences of excessive ROS formation at low irradiances (0.23–0.48 W m^−2^). This is consistent with the suggestion that ROS is the main contributor to UVB-induced adverse effects in plants (Foyer et al. 1994; Takshak and Agrawal 2014). Higher irradiances of UVB (≥ 1 W m^−2^) are proposed to trigger additional toxicity pathways including the destruction of chlorophylls and other photosynthesis-related components, impair energy production in the mitochondria and CPD formation in the nucleus. Demonstration of such causal relationship in several plant species including *Malva parviflora*, *Phaseolus vulgaris*, *Arabidopsis thaliana* (Salama et al. 2011; Li et al. 2015) indicates that these responses are occurring in a diverse set of plants.

This study intended to provide initial effect data to propose a set of putative toxicity pathways relevant for exposure to elevated UVB irradiances in the aquatic plant *L. minor* not acclimated or acclimated to low UVB levels only. As such, it provides only limited information about the temporal changes occurring from onset of the initial acute response to stable compensatory mechanisms and effects occurring under natural PAR, UVA and UVB conditions. The study was also conducted under continuous illumination to limit day and night repair mechanisms and thus accentuate the mode of action (MoA) and adverse effects of UVB. Additional studies to decipher the temporality in such responses under more natural radiation exposure conditions are thus warranted to further characterize the full MoAs of UVB in macrophytes such as *L. minor*.

## Conclusions

The present study has assessed the biological effects of UVB on the aquatic plant *L. minor* at multiple levels of biological organization. Multiple irradiance-dependent toxicity pathways of UVB are proposed, with exposure to low irradiances inducing oxidative stress responses and suppressing photosynthesis, and high irradiances causing CPD formation and disruption of the cellular energy metabolism. The present study has provided novel mechanistic insights into the potential hazards of UVB in aquatic plants by evaluating and proposing linkages between responses occurring at different levels of biological organization. The putative toxicity pathways proposed can further support the development of predictive approaches such as adverse outcome pathways (AOPs) for non-ionizing radiation and serve as a knowledge base for understanding effects of UVB under more ecologically relevant exposure conditions.

### *Author contribution statement*

LX and KET designed the research; LX conducted the research; LX, KAS, YS, and KET analyzed data; BJ measured the light and UVR spectrum; LX and KET drafted the manuscript with contributions from YS, KAS, BJ, and JEO. All authors have read and approved the final manuscript.

## Electronic supplementary material

Below is the link to the electronic supplementary material.

Supplementary file1 (DOCX 1300 kb)

## Abbreviations

Φ_PSII_: Effective PSII efficiency
CPD: Cyclobutane pyridine dimer
EI_50_: 50% effect irradiance
*F*_v_/*F*_m_: Maximal PSII efficiency
LOEI: Lowest observed effect irradiance
LPO: Lipid peroxidation
MMP: Mitochondrial membrane potential
NPQ: Non-photochemical quenching
PAR: Photosynthetically active irradiation
qP: Coefficients of photochemical quenching
ROS: Reactive oxygen species

## References

[CR1] Aarti PD, Tanaka R, Tanaka A (2006). Effects of oxidative stress on chlorophyll biosynthesis in cucumber (*Cucumis sativus*) cotyledons. Physiol Plant.

[CR2] Allen DJ, Nogués S, Baker NR (1998). Ozone depletion and increased UV-B radiation: is there a real threat to photosynthesis?. J Exp Bot.

[CR3] Arambage SC, Grant KM, Pardo I, Ranford-Cartwright L, Hurd H (2009). Malaria ookinetes exhibit multiple markers for apoptosis-like programmed cell death in vitro. Parasites Vectors.

[CR4] Bais AF, Bernhard G, McKenzie RL, Aucamp PJ, Young PJ, Ilyas M, Jöckel P, Deushi M (2019). Ozone–climate interactions and effects on solar ultraviolet radiation. Photochem Photobiol Sci.

[CR5] Bernhard GH, Neale RE, Barnes PW, Neale PJ, Zepp RG, Wilson SR, Andrady AL (2020). Environmental effects of stratospheric ozone depletion, UV radiation and interactions with climate change: UNEP Environmental Effects Assessment Panel, update 2019. Photochem Photobiol Sci.

[CR6] Bornman JF, Barnes PW, Robson TM, Robinson SA, Jansen MAK, Ballaré CL, Flint SD (2019). Linkages between stratospheric ozone, UV radiation and climate change and their implications for terrestrial ecosystems. Photochem Photobiol Sci.

[CR7] Cockell CS, Knowland J (1999). Ultraviolet radiation screening compounds. Biol Rev.

[CR8] Coors A, Kuckelkorn J, Hammers-Wirtz M, Strauss T (2006). Application of in-situ bioassays with macrophytes in aquatic mesocosm studies. Ecotoxicology.

[CR9] Dakora FD, Phillips DA, Adu-Gyamfi JJ (2002). Root exudates as mediators of mineral acquisition in low-nutrient environments. Food security in nutrient-stressed environments: exploiting plants’ genetic capabilities.

[CR10] Dany A-L, Douki T, Triantaphylides C, Cadet J (2001). Repair of the main UV-induced thymine dimeric lesions within *Arabidopsis thaliana* DNA: evidence for the major involvement of photoreactivation pathways. J Photochem Photobiol B.

[CR11] Demmig-Adams B (1990). Carotenoids and photoprotection in plants: a role for the xanthophyll zeaxanthin. Biochim Biophys Acta.

[CR12] Ekperusi AO, Sikoki FD, Nwachukwu EO (2019). Application of common duckweed (*Lemna minor*) in phytoremediation of chemicals in the environment: state and future perspective. Chemosphere.

[CR13] Farooq M, Suresh Babu G, Ray RS, Misra RB, Shankar U, Hans RK (2000). Sensitivity of duckweed (*Lemna major*) to ultraviolet-B radiation. Biochem Biophys Res Commun.

[CR14] Foyer CH, Lelandais M, Kunert KJ (1994). Photooxidative stress in plants. Physiol Plant.

[CR15] Furukawa T, Curtis MJ, Tominey CM, Duong YH, Wilcox BWL, Aggoune D, Hays JB, Britt AB (2010). A shared DNA-damage-response pathway for induction of stem-cell death by UVB and by gamma irradiation. DNA Repair.

[CR16] Gruenhagen RD, Moreland DE (1971). Effects of herbicides on ATP levels in excised soybean hypocotyls. Weed Sci.

[CR17] Habash DZ, Genty B, Baker NR (1994). The consequences of chlorophyll deficiency for photosynthetic light use efficiency in a single nuclear gene mutation of cowpea. Photosynth Res.

[CR18] Hashem HA (2013). Cadmium toxicity induces lipid peroxidation and alters cytokinin content and antioxidant enzyme activities in soybean. Botany.

[CR19] Hideg É, Spetea C, Vass I (1994). Singlet oxygen and free radical production during acceptor- and donor-side-induced photoinhibition: studies with spin trapping EPR spectroscopy. Biochim Biophys Acta.

[CR20] Hideg É, Jansen MAK, Strid Å (2013). UV-B exposure, ROS, and stress: inseparable companions or loosely linked associates?. Trends Plant Sci.

[CR21] Hodges DM, DeLong JM, Forney CF, Prange RK (1999). Improving the thiobarbituric acid-reactive-substances assay for estimating lipid peroxidation in plant tissues containing anthocyanin and other interfering compounds. Planta.

[CR22] Hu Z, Li H, Chen S, Yang Y (2013). Chlorophyll content and photosystem II efficiency in soybean exposed to supplemental ultraviolet-B radiation. Photosynthetica.

[CR23] Jansen MA, Shabala S (2012). 12 Ultraviolet-B radiation: from stressor to regulatory signal. Plant stress physiology.

[CR24] Jansen MAK, Gaba V, Greenburg BM (1998). Higher plants and UV-B radiation: balancing damage, repair and acclimation. Trends Plants Sci.

[CR25] Johnsen B (2020) UV database of Norwegian Radiation and Nuclear Safety Authority (DSA). https://github.com/uvnrpa

[CR26] Jordan BR, James PE, Strid Å, Anthony RG (1994). The effect of ultraviolet-B radiation on gene expression and pigment composition in etiolated and green pea leaf tissue: UV-B-induced changes are gene-specific and dependent upon the developmental stage. Plant Cell Environ.

[CR27] Kaosamphan A, Yamauchi N, Srilaong V, Aiamla-Or S, Wongs-Aree C, Uthairatanakij A (2010). Involvement of chlorophyllase on chlorophyll degradation in stored broccoli florets and its control by UV treatment.

[CR28] Kataria S, Jajoo A, Guruprasad KN (2014). Impact of increasing ultraviolet-B (UV-B) radiation on photosynthetic processes. J Photochem Photobiol B.

[CR29] Kaur N, Sharma I, Kirat K, Pati PK (2016). Detection of reactive oxygen species in *Oryza sativa* L. (Rice). Bio-protocol.

[CR30] Kim KC, Piao MJ, Zheng J, Yao CW, Cha JW, Kumara MHSR, Han X, Kang HK, Lee NH, Hyun JW (2014). Fucodiphlorethol G purified from *Ecklonia cava* suppresses ultraviolet B radiation-induced oxidative stress and cellular damage. Biomol Ther.

[CR31] Klodmann J, Sunderhaus S, Nimtz M, Jänsch L, Braun H-P (2010). Internal architecture of mitochondrial complex I from *Arabidopsis thaliana*. Plant Cell.

[CR32] Krizek DT (2004). Influence of PAR and UV-A in determining plant sensitivity and photomorphogenic responses to UV-B radiation. Photochem Photobiol.

[CR33] Li N, Teranishi M, Yamaguchi H, Matsushita T, Watahiki MK, Tsuge T, Li SS, Hidema J (2015). UVB-induced CPD photolyase gene expression is regulated by UVR8-dependent and -independent pathways in Arabidopsis. Plant Cell Physiol.

[CR34] Lidon FC, Ramalho JC (2011). Impact of UV-B irradiation on photosynthetic performance and chloroplast membrane components in *Oryza sativa* L. J Photochem Photobiol B.

[CR35] Livingston AK, Cruz JA, Kohzuma K, Dhingra A, Kramer DM (2010). An *Arabidopsis* mutant with high cyclic electron flow around photosystem I (hcef) involving the NADPH dehydrogenase complex. Plant Cell.

[CR36] Livingston AK, Kanazawa A, Cruz JA, Kramer DM (2010). Regulation of cyclic electron flow in C3 plants: differential effects of limiting photosynthesis at ribulose-1,5-bisphosphate carboxylase/oxygenase and glyceraldehyde-3-phosphate dehydrogenase. Plant Cell Environ.

[CR37] Lodish H, Berk A, Zipursky SL, Matsudaira P, Baltimore D, Darnell J (2000) Section 16.2, Electron transport and oxidative phosphorylation. In: Molecular cell biology, 4th edn. W. H. Freeman, New York. https://www.ncbi.nlm.nih.gov/books/NBK21528/

[CR38] Lois R (1994). Accumulation of UV-absorbing flavonoids induced by UV-B radiation in *Arabidopsis thaliana* L. Planta.

[CR39] Løvdal T, Olsen KM, Slimestad R, Verheul M, Lillo C (2010). Synergetic effects of nitrogen depletion, temperature, and light on the content of phenolic compounds and gene expression in leaves of tomato. Phytochemistry.

[CR40] Lubos E, Loscalzo J, Handy DE (2011). Glutathione peroxidase-1 in health and disease: from molecular mechanisms to therapeutic opportunities. Antioxid Redox Signal.

[CR41] Lytvyn DI, Yemets AI, Blume YB (2010). UV-B overexposure induces programmed cell death in a BY-2 tobacco cell line. Environ Exp Bot.

[CR42] Manova V, Gruszka D (2015). DNA damage and repair in plants—from models to crops. Front Plant Sci.

[CR43] Marwood CA, Greenberg BM (1996). Effect of supplementary UVB radiation on chlorophyll synthesis and accumulation of photosystems during chloroplast development in *Spirodela oligorrhiza*. Photochem Photobiol.

[CR44] Maxwell K, Johnson GN (2000). Chlorophyll fluorescence—a practical guide. J Exp Bot.

[CR450] Mayer B, Kylling A (2005). Technical note: the libRadtran software package for radiative transfer calculations - description and examples of use. Atmos Chem Phys.

[CR45] McLay CL (1976). The effect of pH on the population growth of three species of duckweed: *Spirodela oligorrhiza*, *Lemna minor* and *Wolffia arrhiza*. Freshw Biol.

[CR46] Mittler R (2002). Oxidative stress, antioxidants and stress tolerance. Trends Plant Sci.

[CR47] Moody M, Miller J, Blaise C, Férard JF (2005). *Lemna minor* growth inhibition test. Small-scale freshwater toxicity investigations.

[CR48] Moon YR, Lee MH, Tovuu A, Lee C-H, Chung BY, Park Y-I, Kim J-H (2011). Acute exposure to UV-B sensitizes cucumber, tomato, and Arabidopsis plants to photooxidative stress by inhibiting thermal energy dissipation and antioxidant defense. J Radiat Res.

[CR49] Mori J, Smith R (2019). Transmission of waterborne fish and plant pathogens in aquaponics and their control with physical disinfection and filtration: a systematized review. Aquaculture.

[CR50] Mosadegh H, Trivellini A, Lucchesini M, Ferrante A, Maggini R, Vernieri P, Sodi AM (2019). UV-B physiological changes under conditions of distress and eustress in sweet basil. Plants.

[CR51] Müller P, Li X-P, Niyogi KK (2001). Non-photochemical quenching. A response to excess light energy. Plant Physiol.

[CR52] Nawkar GM, Maibam P, Park JH, Sahi VP, Lee SY, Kang CH (2013). UV-induced cell death in plants. Int J Mol Sci.

[CR53] NCAR U (2018) Quick TUV calculator [WWW Document]. https://cprm.acom.ucar.edu/Models/TUV/Interactive_TUV/

[CR54] OECD (2006) Test No. 221: *Lemna* sp. growth inhibition test. 10.1787/9789264016194-en

[CR55] Ohnishi N, Allakhverdiev SI, Takahashi S, Higashi S, Watanabe M, Nishiyama Y, Murata N (2005). Two-step mechanism of photodamage to photosystem II: step 1 occurs at the oxygen-evolving complex and step 2 occurs at the photochemical reaction center. Biochemistry.

[CR56] Oxborough K, Baker NR (1997). Resolving chlorophyll a fluorescence images of photosynthetic efficiency into photochemical and non-photochemical components—calculation of qP and Fv-/Fm-; without measuring Fo. Photosynth Res.

[CR57] Pfaffl MW (2001). A new mathematical model for relative quantification in real-time RT-PCR. Nucleic Acids Res.

[CR58] Prasad SM, Dwivedi R, Zeeshan M (2005). Growth, photosynthetic electron transport, and antioxidant responses of young soybean seedlings to simultaneous exposure of nickel and UV-B stress. Photosynthetica.

[CR59] Radić S, Stipaničev D, Cvjetko P, Rajčić MM, Širac S, Pevalek-Kozlina B, Pavlica M (2011). Duckweed *Lemna minor* as a tool for testing toxicity and genotoxicity of surface waters. Ecotoxicol Environ Saf.

[CR60] Rastogi RP, Richa KA, Tyagi MB, Sinha RP (2010). Molecular mechanisms of ultraviolet radiation-induced DNA damage and repair. J Nucleic Acids.

[CR61] Razinger J, Drinovec L, Zrimec A (2010). Real-time visualization of oxidative stress in a floating macrophyte *Lemna minor* L. exposed to cadmium, copper, menadione, and AAPH. Environ Toxicol.

[CR62] Rivas-García T, González-Estrada RR, Chiquito-Contreras RG, Reyes-Pérez JJ, González-Salas U, Hernández-Montiel LG, Murillo-Amador B (2020). Biocontrol of phytopathogens under aquaponics systems. Water.

[CR63] Roro AG, Dukker SAF, Melby TI, Solhaug KA, Torre S, Olsen JE (2017). UV-B-induced inhibition of stem elongation and leaf expansion in pea depends on modulation of gibberellin metabolism and intact gibberellin signalling. J Plant Growth Regul.

[CR64] Rozema J, Jvd S, Björn LO, Caldwell M (1997). UV-B as an environmental factor in plant life: stress and regulation. Trends Ecol Evol.

[CR65] Ruban AV (2016). Nonphotochemical chlorophyll fluorescence quenching: mechanism and effectiveness in protecting plants from photodamage. Plant Physiol.

[CR66] Salama HMH, Watban AAA, Al-Fughom AT (2011). Effect of ultraviolet radiation on chlorophyll, carotenoid, protein and proline contents of some annual desert plants. Saudi J Biol Sci.

[CR67] Schmidt ÉC, Pereira B, Pontes CLM, Santos Rd, Scherner F, Horta PA, Martins RdP, Latini A, Maraschin M, Bouzon ZL (2012). Alterations in architecture and metabolism induced by ultraviolet radiation-B in the carragenophyte *Chondracanthus teedei* (Rhodophyta, Gigartinales). Protoplasma.

[CR68] Shi C, Yang F, Zhu X, Du E, Yang Y, Wang S, Wu Q, Zhang Y (2016). Evaluation of housekeeping genes for quantitative real-time PCR analysis of *Bradysia odoriphaga* (Diptera: Sciaridae). Int J Mol Sci.

[CR69] Song Y, Rundberget JT, Evenseth LM, Xie L, Gomes T, Høgåsen T, Iguchi T, Tollefsen KE (2016). Whole-organism transcriptomic analysis provides mechanistic insight into the acute toxicity of emamectin benzoate in *Daphnia magna*. Environ Sci Technol.

[CR70] Stouvenakers G, Dapprich P, Massart S, Jijakli MH, Goddek S, Joyce A, Kotzen B, Burnell G (2019). Plant pathogens and control strategies in aquaponics. Aquaponics food production systems.

[CR71] Strid Å, Porra RJ (1992). Alterations in pigment content in leaves of *Pisum sativum* after exposure to supplementary UV-B. Plant Cell Physiol.

[CR72] Strid Å, Chow WS, Anderson JM (1994). UV-B damage and protection at the molecular level in plants. Photosynth Res.

[CR73] Suthaparan A, Solhaug KA, Stensvand A, Gislerød HR (2017). Daily light integral and day light quality: potentials and pitfalls of nighttime UV treatments on cucumber powdery mildew. J Photochem Photobiol B.

[CR74] Szőllősi E, Veres S, Kanalas P, Oláh V, Solti Á, Sarvari E, Meszaros I (2008). Effects of UV-B radiation and water stress on chlorophyll fluorescence parameters and activity of xanthophyll cycle in leaves of sessile oak (*Quercus petraea*) seedlings. Acta Biol Szeged.

[CR75] Takshak S, Agrawal SB (2014). Effect of ultraviolet-B radiation on biomass production, lipid peroxidation, reactive oxygen species, and antioxidants in *Withania somnifera*. Biol Plant.

[CR76] Talukdar M, Shahjahan M, Rahman (2013). Suitability of duckweed (*Lemna minor*) as feed for fish in polyculture system. Int J Agric Res Innov Technol.

[CR77] Tang X, Zhang N, Si H, Calderón-Urrea A (2017). Selection and validation of reference genes for RT-qPCR analysis in potato under abiotic stress. Plant Methods.

[CR78] Teardo E, Carraretto L, Bortoli SD, Costa A, Behera S, Wagner R, Schiavo FL, Formentin E, Szabo I (2015). Alternative splicing-mediated targeting of the Arabidopsis GLUTAMATE RECEPTOR3.5 to mitochondria affects organelle morphology. Plant Physiol.

[CR79] Teramura AH, Worrest RC, Caldwell MM (1986). Interaction between UV-B radiation and other stresses in plants. Stratospheric ozone reduction, solar ultraviolet radiation and plant life.

[CR80] Teramura AH, Sullivan JH (1994). Effects of UV-B radiation on photosynthesis and growth of terrestrial plants. Photosynth Res.

[CR81] Teranishi M, Iwamatsu Y, Hidema J, Kumagai T (2004). Ultraviolet-B sensitivities in Japanese lowland rice cultivars: cyclobutane pyrimidine dimer photolyase activity and gene mutation. Plant Cell Physiol.

[CR82] Tikhonov AN (2017). Photosynthetic electron and proton transport in chloroplasts: EPR Study of ΔpH generation, an overview. Cell Biochem Biophys.

[CR83] Ulm R, Jenkins GI (2015). Q&A: how do plants sense and respond to UV-B radiation?. BMC Biol.

[CR84] Van Os EA, Bruins M, Postma J, Willemsen-de Klein MJEIM (2004). Investigations on crop developments and microbial suppressiveness of *Pythium aphanidermatum* after different disinfection treatments of the circulating nutrient solution. Acta Hortic.

[CR85] Velichkova KN, Sirakov IN (2013). The usage of aquatic floating macrophytes (*Lemna* and *Wolffia*) as biofilter in recirculation aquaculture system (RAS). Turk J Fish Aquat Sci.

[CR86] Wellburn AR (1994). The spectral determination of chlorophylls *a* and *b*, as well as total carotenoids, using various solvents with spectrophotometers of different resolution. J Plant Physiol.

[CR87] Whitehead RF, De Mora SJ, Demers S, Vernet M, Demers S, De Mora S (2000). Enhanced UV radiation—a new problem for the marine environment. The effects of UV radiation in the marine environment.

[CR88] Williamson CE, Neale PJ, Hylander S, Rose KC, Figueroa FL, Robinson SA, Häder D-P, Wängberg S-Å, Worrest RC (2019). The interactive effects of stratospheric ozone depletion, UV radiation, and climate change on aquatic ecosystems. Photochem Photobiol Sci.

[CR89] Wilson MI, Ghosh S, Gerhardt KE, Holland N, Babu TS, Edelman M, Dumbroff EB, Greenberg BM (1995). In vivo photomodification of ribulose-1,5-bisphosphate carboxylase/oxygenase holoenzyme by ultraviolet-B radiation (formation of a 66-kilodalton variant of the large subunit). Plant Physiol.

[CR90] Xie L, Gomes T, Solhaug KA, Song Y, Tollefsen KE (2018). Linking mode of action of the model respiratory and photosynthesis uncoupler 3,5-dichlorophenol to adverse outcomes in *Lemna minor*. Aquat Toxicol.

[CR91] Xue L, Li S, Sheng H, Feng H, Xu S, An L (2007). Nitric oxide alleviates oxidative damage induced by enhanced ultraviolet-B radiation in cyanobacterium. Curr Microbiol.

[CR92] Zezulka Š, Kummerová M, Babula P, Váňová L (2013). *Lemna minor* exposed to fluoranthene: growth, biochemical, physiological and histochemical changes. Aquat Toxicol.

