# Supplementary material for: Effects of artificial ultraviolet B radiation on the macrophyte Lemna minor: a conceptual study for toxicity pathway characterization
Source: Planta. 2020 Oct 14;252(5):86. doi: 10.1007/s00425-020-03482-3 (PMC7560917; doi:10.1007/s00425-020-03482-3)
Supplement: Supplementary file 1 — Supplementary file1 (DOCX 1300 kb) [file 425_2020_3482_MOESM1_ESM.docx]

**Effects of artificial ultraviolet B radiation on the macrophyte *Lemna minor:* a conceptual study for toxicity pathway characterization**

**Li Xie^1, 3^, Knut Asbjørn Solhaug^2, 3^, You Song^1, 3^, Bjørn Johnsen^3, 4^, Jorunn Elisabeth Olsen ^3,5^, Knut Erik Tollefsen^1, 2, 3*^**

^1^ Section of Ecotoxicology and Risk Assessment, Norwegian Institute for Water Research (NIVA), Gaustadalléen 21, N-0349 Oslo, Norway

^2^ Faculty of Environmental Sciences and Natural Resource Management, Norwegian University of Life Sciences (NMBU), P.O. Box 5003, N-1432 Ås, Norway

^3^ Centre for Environmental Radioactivity, Norwegian University of Life Sciences (NMBU), Post box 5003, N-1432 Ås, Norway

^4^ Norwegian Radiation and Nuclear Safety Authority (DSA), 1361 Østerås, Norway

^5^ Faculty of Biosciences, Institute of Plant Sciences, Norwegian University of Life Sciences (NMBU), P.O. Box 5003, N-1432 Ås, Norway

**Corresponding author:**

Knut Erik [Tollefsen](https://www.google.no/url?sa=t&rct=j&q=&esrc=s&source=web&cd=1&ved=0ahUKEwju4ty69sXMAhWEVywKHQHMAVEQFggcMAA&url=http%3A%2F%2Fwww.niva.no%2Fse-ansatt%3Fnavn%3DKnut-Erik%2520Tollefsen&usg=AFQjCNEcD7iL_TI4IPB7RrXApxFvdea9ZA&sig2=W3HomTsh5RonGjOyuROqOQ), Norwegian Institute for Water Research (NIVA), Section of Ecotoxicology and Risk Assessment, Gaustadalléen 21, N-0349 OSLO, Norway; Tel: (+47) 40556070, Fax: (+47) 221852 00; E-mail address: [KET@niva.no](mailto:KET@niva.no)

**Acknowledgements**

Funding from the Research Council of Norway through the Center of Excellence funding scheme, project number 223268, and NIVA’s Computational Toxicology Program, NCTP ([www.niva.no/nctp](http://www.niva.no/nctp)) through the strategic institute initiative “Digi-SIS” is gratefully acknowledged.

**Suppl. Table S1** Primers used for qRT-PCR analyses of transcript levels in *Lemna minor* exposed to UV radiation

| **Function** | **Gene name** | **Gene symbol** | **Forward primer (5'-3')** | **Reverse primer (5'-3')** | **Amplicon size (BP)** | **Annealing temperature (°C)** | **Amplification efficiency (%)** | **BLAST hit (green plants)** | **Hit accession** | **Sequence length** | **E-Value** | **Similarity** | **Bit-Score** | **Alignment length** | **Positives** |
| --- | --- | --- | --- | --- | --- | --- | --- | --- | --- | --- | --- | --- | --- | --- | --- |
| Housekeeping gene | S18 | *RPS18* | ATCCACTCTCACCGTGGTCT | CGGTGGTCTTCGAGTGTTGA | 70 | 53.8 | 93.5 | ribosomal protein S18 [Acyrthosiphon pisum]gi | NP_001119689, XP_015363554, | 473 | 1.23E-78 | 90.196078 | 236.113 | 153 | 138 |
| Housekeeping gene | elongation factor 1-alpha | *EF1a* | TATCAAGCCCGGAATGGTGG | TGCATCTCCACGGACTTCAC | 72 | 55.1 | 96.8 | elongation factor 1 alpha [Actinidia deliciosa] | AAX54511 | 1747 | 0.00E+00 | 99.313501 | 894.034 | 437 | 434 |
| Housekeeping gene | glyceraldehyde-3-phosphate dehydrogenase | *GAPDH* | TTGGTACGACAACGAGTGGG | CACTGTCGATTACTGGGCGT | 82 | 53.8 | 105.2 | PREDICTED: glyceraldehyde-3-phosphate dehydrogenase 2, cytosolic [Oryza brachyantha] | XP_006652435 | 1195 | 0.00E+00 | 96.716418 | 607.446 | 335 | 324 |
| antioxidant enzyme | peroxidase dismutase | *SOD1* | CCTGAAGCCTCCTCCTTACG | CCAGTGGAACTCCAGCGTCT | 73 | 55.1 | 101.4 | superoxide dismutase [Monoraphidium neglectum]gi | XP_013895475, KIY96455 | 256 | 2.52E-21 | 89.361702 | 87.4261 | 47 | 42 |
| antioxidant enzyme | glutathione peroxidase | *GPX* | CGCCTGTACCCTACCTTGTG | CACTCATAGCGCTCTCGTCC | 93 | 55.1 | 102.4 | glutathione peroxidase [Monoraphidium neglectum]gi | XP_013900345, KIZ01326 | 696 | 3.40E-75 | 78.857143 | 232.261 | 175 | 138 |
| UV-induced DNA-damage | dna-damage-repair toleration protein | *DRT111* | GCGGGTGCTGATATTCGAGA | GTCTCCTCTGCCCGTTCAAA | 90 | 53.8 | 92.3 | PREDICTED: DNA-damage-repair/toleration protein DRT111, chloroplastic [Vitis vinifera] | XP_010655737 | 1518 | 1.77E-137 | 82.908163 | 408.297 | 392 | 325 |
| DNA damage repair | dna repair protein rad50 | *RAD50* | AGGGCAAGAGAACTTCCAGC | CGTGCTGTCGTTGACCAATC | 77 | 53.8 | 101.6 | PREDICTED: DNA repair protein RAD50 isoform X1 [Elaeis guineensis] | XP_010927015 | 4064 | 0.00E+00 | 90.114068 | 2026.52 | 1315 | 1185 |
| DNA double-strand break sensor | serine threonine-protein kinase atm | *ATM* | ACAGCTCATTCAGGACGCAA | TCACCTTTGCGGACCTCATC | 94 | 53.8 | 98.5 | PREDICTED: serine/threonine-protein kinase ATM isoform X2 [Elaeis guineensis] | XP_019710063 | 3892 | 0.00E+00 | 82.982172 | 1793.86 | 1234 | 1024 |
| apoptosis | apoptosis inhibitor 5-like | *API5* | AAGCTGCCACCAAGCAAAAC | TTTCTTCGGGGGTTGACCTG | 75 | 53.8 | 94.3 | PREDICTED: apoptosis inhibitor 5-like protein API5 [Elaeis guineensis] | XP_010904879 | 1757 | 0.00E+00 | 83.736264 | 674.855 | 455 | 381 |
| positive regulator of cell death | metacaspase-1 | *AMC1* | GGTGGCGAGGTCATTTCCTT | GTCATCACCCCTGTTGAGGT | 95 | 53.8 | 103.5 | PREDICTED: metacaspase-1 [Nicotiana tabacum] | XP_016501312 | 384 | 2.76E-69 | 93.965517 | 218.394 | 116 | 109 |
| ETC/apoptosis | cytochrome c | *CYC* | TACCTGAAGAGTTCGACGGC | TCAGATGGCGGAGAACAACC | 100 | 53.8 | 96.7 | cytochrome c [Vigna radiata var. radiata]gi | XP_014496567, XP_017416997, KOM36983, BAT83489 | 614 | 6.28E-73 | 94.642857 | 222.246 | 112 | 106 |
| photophosphorylation | atp synthase gamma | *ATPC1* | GCCATTGCCGACGATGTTTT | TTCACCAGCGACACGAACTT | 89 | 53.8 | 98.7 | PREDICTED: ATP synthase gamma chain, chloroplastic [Nelumbo nucifera] | XP_010276111 | 2299 | 0.00E+00 | 90.4 | 594.349 | 375 | 339 |
| CO2 fixing | ribulose bisphosphate carboxylase | *RBSC* | CAGTGCATCAGCTTCATCGC | GCTTCAATGGGGACGGAGAA | 89 | 53.8 | 97.2 | RBS6_LEMGIRecName: Full=Ribulose bisphosphate carboxylase small chain SSU5B, chloroplastic; Short=RuBisCO small subunit SSU5B; Flags: Precursorgi | P19312, CAA35100 | 1090 | 5.46E-108 | 98.113208 | 319.701 | 159 | 156 |
| glycolysis | pyruvate kinase | *PK* | TCAGAGACCTTCCGAGGCAA | CGCATAGTTGTCGAAGACGG | 93 | 53.8 | 105.8 | pyruvate kinase [Volvox carteri f. nagariensis]gi | XP_002954743, EFJ44149 | 323 | 1.09E-40 | 82.291667 | 147.132 | 96 | 79 |
| oxdative phosphorylation | nadh dehydrogenase | *NDUFV1* | TGTGGAGGAGTGAGAGGAGG | GGAGAGGAACCGAAGAACCG | 70 | 55.1 | 96.2 | hypothetical protein AQUCO_04000034v1 [Aquilegia coerulea]gi | PIA33693, PIA33695, PIA33696 | 1892 | 0.00E+00 | 95.670103 | 892.878 | 485 | 464 |
| chlorophyll synthesis | chlorophyllase | *CLH2* | CGCGACGATCTGCTTACTCT | TCAAAGTCAGCGGTGGAGAC | 71 | 53.8 | 103.5 | chlorophyllase-2, chloroplastic [Cinnamomum micranthum f. kanehirae] | RWR94759 | 1033 | 1.20E-117 | 74 | 348.591 | 300 | 222 |
| Carotenoid biosynthesis | phytoene synthase | *PSY2* | TCGCTGCTGCTTTATCGGAA | AGCTCGCTTGGTGAAGTTGT | 72 | 53.8 | 97.1 | phytoene synthase 2, chloroplastic [Camellia sinensis]gi | XP_028094815, AJB84620 | 1969 | 0.00E+00 | 90.988372 | 593.578 | 344 | 313 |
| Flavonoid biosynthesis | flavonoid 3 -monooxygenase-like | *CYP75B* | TGGTGAAGGAGACACTTCGC | TACCCAGCTATGGAGCAGGT | 85 | 53.8 | 96.6 | Flavonoid 3'-monooxygenase [Vitis vinifera] | RVW90623 | 1592 | 1.14E-115 | 68.470588 | 358.607 | 425 | 291 |
| D1 protein synthesis (PSII) | photosystem ii protein d1 | *PSBA* | GCTCACGGTTATTTTGGCCG | GGCCAAGCAGCTAGGAAGAA | 86 | 53.8 | 105.5 | photosystem II Q(b) protein D1, partial (chloroplast) [Cypripedium japonicum] | BBD13759 | 1175 | 0.00E+00 | 98.913043 | 701.049 | 368 | 364 |

**Suppl. Table S2** Pearson correlation matrix obtained from all the biological endpoints determined in *Lemna minor* exposed to UV radiation for 7 days.

| Variables | FN | FA | DW | ROS | CPD | MMP | LPO | Fv/Fm | ΦPSII | qP | NPQ | Chl a | Chl b | Car | *PsbA* | *SOD* | *GPx* | *DRT111* | *DUFV1* | *ATPC1* | *PK* | *RBSC* | *RAD50* | *API* | *ATM* | *AMC1* | *CYC* | *CHL2* | *PSY2* | *CYP75B* |
| --- | --- | --- | --- | --- | --- | --- | --- | --- | --- | --- | --- | --- | --- | --- | --- | --- | --- | --- | --- | --- | --- | --- | --- | --- | --- | --- | --- | --- | --- | --- |
| FN | **1** | **0.976** | **0.988** | -0.648 | **-0.974** | **0.989** | **-0.84** | **0.954** | **0.976** | **0.944** | -0.254 | **0.993** | **0.978** | -0.284 | 0.722 | -0.458 | **-0.853** | -0.159 | 0.687 | 0.438 | **0.838** | -0.462 | -0.694 | -0.306 | -0.13 | -0.071 | 0.704 | **0.879** | **0.833** | 0.045 |
| FA | **0.976** | **1** | **0.963** | -0.751 | **-0.982** | **0.988** | **-0.912** | **0.877** | **0.974** | **0.97** | -0.361 | **0.965** | **0.982** | -0.383 | **0.785** | -0.474 | **-0.845** | -0.119 | 0.688 | 0.486 | **0.898** | -0.351 | **-0.764** | -0.41 | -0.224 | -0.126 | 0.681 | **0.895** | **0.882** | 0.016 |
| DW | **0.988** | **0.963** | **1** | -0.557 | **-0.946** | **0.986** | **-0.788** | **0.947** | **0.936** | **0.904** | -0.154 | **0.984** | **0.962** | -0.178 | 0.665 | -0.329 | **-0.806** | -0.009 | 0.605 | 0.377 | **0.818** | -0.386 | -0.6 | -0.222 | -0.068 | 0.064 | 0.617 | **0.831** | **0.863** | 0.181 |
| ROS | -0.648 | -0.751 | -0.557 | **1** | **0.772** | -0.673 | **0.919** | -0.475 | **-0.787** | **-0.84** | **0.763** | -0.631 | -0.725 | **0.794** | **-0.879** | **0.816** | 0.656 | 0.488 | **-0.791** | -0.649 | **-0.825** | 0.298 | **0.931** | 0.69 | 0.415 | 0.569 | -0.711 | **-0.829** | -0.615 | 0.444 |
| CPD | **-0.974** | **-0.982** | **-0.946** | **0.772** | **1** | **-0.97** | **0.927** | **-0.885** | **-0.984** | **-0.981** | 0.366 | **-0.979** | **-0.988** | 0.401 | **-0.822** | 0.576 | **0.849** | 0.223 | **-0.792** | -0.555 | **-0.908** | 0.476 | 0.752 | 0.333 | 0.119 | 0.122 | **-0.791** | **-0.953** | **-0.834** | 0.047 |
| MMP | **0.989** | **0.988** | **0.986** | -0.673 | **-0.97** | **1** | **-0.868** | **0.914** | **0.967** | **0.942** | -0.244 | **0.978** | **0.987** | -0.272 | 0.727 | -0.417 | **-0.843** | -0.094 | 0.649 | 0.392 | **0.863** | -0.4 | -0.712 | -0.353 | -0.157 | -0.052 | 0.633 | **0.87** | **0.904** | 0.125 |
| LPO | **-0.84** | **-0.912** | **-0.788** | **0.919** | **0.927** | **-0.868** | **1** | -0.661 | **-0.907** | **-0.926** | 0.487 | **-0.835** | **-0.916** | 0.521 | **-0.829** | 0.649 | **0.842** | 0.339 | **-0.766** | -0.523 | **-0.88** | 0.437 | **0.87** | 0.535 | 0.296 | 0.325 | -0.735 | **-0.909** | **-0.807** | 0.158 |
| Fv/Fm | **0.954** | **0.877** | **0.947** | -0.475 | **-0.885** | **0.914** | -0.661 | **1** | **0.91** | **0.86** | -0.182 | **0.95** | **0.881** | -0.215 | 0.642 | -0.42 | -0.728 | -0.14 | 0.651 | 0.42 | 0.741 | -0.463 | -0.551 | -0.159 | 0.005 | 0.015 | 0.669 | **0.8** | 0.711 | 0.057 |
| ΦPSII | **0.976** | **0.974** | **0.936** | **-0.787** | **-0.984** | **0.967** | **-0.907** | **0.91** | **1** | **0.984** | -0.422 | **0.965** | **0.972** | -0.458 | **0.825** | -0.625 | **-0.848** | -0.3 | **0.787** | 0.536 | **0.891** | -0.478 | **-0.813** | -0.431 | -0.199 | -0.229 | **0.77** | **0.934** | **0.817** | -0.106 |
| qP | **0.944** | **0.97** | **0.904** | **-0.84** | **-0.981** | **0.942** | **-0.926** | **0.86** | **0.984** | **1** | -0.527 | **0.941** | **0.951** | -0.558 | **0.899** | -0.658 | **-0.784** | -0.247 | **0.829** | 0.651 | **0.948** | -0.374 | **-0.813** | -0.428 | -0.185 | -0.219 | **0.792** | **0.959** | **0.81** | -0.159 |
| NPQ | -0.254 | -0.361 | -0.154 | **0.763** | 0.366 | -0.244 | 0.487 | -0.182 | -0.422 | -0.527 | **1** | -0.226 | -0.253 | **0.995** | -0.731 | 0.746 | 0.162 | 0.387 | -0.593 | **-0.786** | -0.556 | -0.15 | 0.676 | 0.619 | 0.449 | 0.654 | -0.511 | -0.484 | -0.139 | **0.761** |
| Chl *a* | **0.993** | **0.965** | **0.984** | -0.631 | **-0.979** | **0.978** | **-0.835** | **0.95** | **0.965** | **0.941** | -0.226 | **1** | **0.977** | -0.261 | 0.737 | -0.466 | **-0.827** | -0.138 | 0.726 | 0.479 | **0.852** | -0.488 | -0.641 | -0.21 | -0.027 | 0.006 | 0.742 | **0.904** | **0.823** | 0.065 |
| Chl *b* | **0.978** | **0.982** | **0.962** | -0.725 | **-0.988** | **0.987** | **-0.916** | **0.881** | **0.972** | **0.951** | -0.253 | **0.977** | **1** | -0.289 | **0.755** | -0.491 | **-0.873** | -0.181 | 0.717 | 0.425 | **0.874** | -0.495 | -0.733 | -0.337 | -0.121 | -0.077 | 0.705 | **0.914** | **0.888** | 0.079 |
| Car | -0.284 | -0.383 | -0.178 | **0.794** | 0.401 | -0.272 | 0.521 | -0.215 | -0.458 | -0.558 | **0.995** | -0.261 | -0.289 | **1** | **-0.768** | **0.804** | 0.19 | 0.442 | -0.653 | **-0.798** | -0.587 | -0.073 | 0.703 | 0.613 | 0.407 | 0.656 | -0.557 | -0.533 | -0.167 | **0.757** |
| *PSBA* | 0.722 | **0.785** | 0.665 | **-0.879** | **-0.822** | 0.727 | **-0.829** | 0.642 | **0.825** | **0.899** | -0.731 | 0.737 | **0.755** | **-0.768** | **1** | **-0.801** | -0.483 | -0.252 | **0.914** | **0.841** | **0.957** | -0.207 | -0.742 | -0.38 | -0.046 | -0.231 | **0.771** | **0.925** | 0.664 | -0.302 |
| *SOD* | -0.458 | -0.474 | -0.329 | **0.816** | 0.576 | -0.417 | 0.649 | -0.42 | -0.625 | -0.658 | 0.746 | -0.466 | -0.491 | **0.804** | **-0.801** | **1** | 0.422 | 0.753 | **-0.896** | -0.727 | -0.63 | 0.502 | 0.736 | 0.436 | 0.116 | 0.579 | **-0.812** | -0.745 | -0.255 | 0.655 |
| *GPX* | **-0.853** | **-0.845** | **-0.806** | 0.656 | **0.849** | **-0.843** | **0.842** | -0.728 | **-0.848** | **-0.784** | 0.162 | **-0.827** | **-0.873** | 0.19 | -0.483 | 0.422 | **1** | 0.428 | -0.517 | -0.185 | -0.585 | 0.643 | **0.762** | 0.477 | 0.408 | 0.351 | -0.648 | -0.712 | -0.662 | 0.095 |
| *DRT111* | -0.159 | -0.119 | -0.009 | 0.488 | 0.223 | -0.094 | 0.339 | -0.14 | -0.3 | -0.247 | 0.387 | -0.138 | -0.181 | 0.442 | -0.252 | 0.753 | 0.428 | **1** | -0.481 | -0.197 | -0.075 | 0.708 | 0.567 | 0.455 | 0.315 | **0.774** | -0.543 | -0.312 | 0.123 | 0.685 |
| *NDUFV1* | 0.687 | 0.688 | 0.605 | **-0.791** | **-0.792** | 0.649 | **-0.766** | 0.651 | **0.787** | **0.829** | -0.593 | 0.726 | 0.717 | -0.653 | **0.914** | **-0.896** | -0.517 | -0.481 | **1** | **0.822** | **0.836** | -0.524 | -0.648 | -0.187 | 0.139 | -0.217 | **0.921** | **0.933** | 0.495 | -0.383 |
| *ATPC1* | 0.438 | 0.486 | 0.377 | -0.649 | -0.555 | 0.392 | -0.523 | 0.42 | 0.536 | 0.651 | **-0.786** | 0.479 | 0.425 | **-0.798** | **0.841** | -0.727 | -0.185 | -0.197 | **0.822** | **1** | 0.718 | -0.038 | -0.433 | -0.095 | 0.083 | -0.168 | **0.782** | 0.709 | 0.222 | -0.564 |
| *PK* | **0.838** | **0.898** | **0.818** | **-0.825** | **-0.908** | **0.863** | **-0.88** | 0.741 | **0.891** | **0.948** | -0.556 | **0.852** | **0.874** | -0.587 | **0.957** | -0.63 | -0.585 | -0.075 | **0.836** | 0.718 | **1** | -0.201 | -0.713 | -0.333 | -0.026 | -0.066 | 0.705 | **0.945** | **0.835** | -0.06 |
| *RBSC* | -0.462 | -0.351 | -0.386 | 0.298 | 0.476 | -0.4 | 0.437 | -0.463 | -0.478 | -0.374 | -0.15 | -0.488 | -0.495 | -0.073 | -0.207 | 0.502 | 0.643 | 0.708 | -0.524 | -0.038 | -0.201 | **1** | 0.352 | 0.001 | -0.143 | 0.189 | -0.622 | -0.495 | -0.204 | 0.112 |
| *RAD50* | -0.694 | **-0.764** | -0.6 | **0.931** | 0.752 | -0.712 | **0.87** | -0.551 | **-0.813** | **-0.813** | 0.676 | -0.641 | -0.733 | 0.703 | -0.742 | 0.736 | **0.762** | 0.567 | -0.648 | -0.433 | -0.713 | 0.352 | **1** | **0.846** | 0.613 | 0.703 | -0.6 | -0.731 | -0.62 | 0.413 |
| *API* | -0.306 | -0.41 | -0.222 | 0.69 | 0.333 | -0.353 | 0.535 | -0.159 | -0.431 | -0.428 | 0.619 | -0.21 | -0.337 | 0.613 | -0.38 | 0.436 | 0.477 | 0.455 | -0.187 | -0.095 | -0.333 | 0.001 | **0.846** | **1** | **0.881** | **0.843** | -0.13 | -0.27 | -0.355 | 0.408 |
| *ATM* | -0.13 | -0.224 | -0.068 | 0.415 | 0.119 | -0.157 | 0.296 | 0.005 | -0.199 | -0.185 | 0.449 | -0.027 | -0.121 | 0.407 | -0.046 | 0.116 | 0.408 | 0.315 | 0.139 | 0.083 | -0.026 | -0.143 | 0.613 | **0.881** | **1** | **0.802** | 0.039 | 0.029 | -0.089 | 0.433 |
| *AMC1* | -0.071 | -0.126 | 0.064 | 0.569 | 0.122 | -0.052 | 0.325 | 0.015 | -0.229 | -0.219 | 0.654 | 0.006 | -0.077 | 0.656 | -0.231 | 0.579 | 0.351 | **0.774** | -0.217 | -0.168 | -0.066 | 0.189 | 0.703 | **0.843** | **0.802** | **1** | -0.266 | -0.133 | 0.093 | **0.774** |
| *CYC* | 0.704 | 0.681 | 0.617 | -0.711 | **-0.791** | 0.633 | -0.735 | 0.669 | **0.77** | **0.792** | -0.511 | 0.742 | 0.705 | -0.557 | **0.771** | **-0.812** | -0.648 | -0.543 | **0.921** | **0.782** | 0.705 | -0.622 | -0.6 | -0.13 | 0.039 | -0.266 | **1** | **0.87** | 0.355 | -0.496 |
| *CHL2* | **0.879** | **0.895** | **0.831** | **-0.829** | **-0.953** | **0.87** | **-0.909** | **0.8** | **0.934** | **0.959** | -0.484 | **0.904** | **0.914** | -0.533 | **0.925** | -0.745 | -0.712 | -0.312 | **0.933** | 0.709 | **0.945** | -0.495 | -0.731 | -0.27 | 0.029 | -0.133 | **0.87** | **1** | 0.752 | -0.16 |
| *PSY2* | **0.833** | **0.882** | **0.863** | -0.615 | **-0.834** | **0.904** | **-0.807** | 0.711 | **0.817** | **0.81** | -0.139 | **0.823** | **0.888** | -0.167 | 0.664 | -0.255 | -0.662 | 0.123 | 0.495 | 0.222 | **0.835** | -0.204 | -0.62 | -0.355 | -0.089 | 0.093 | 0.355 | 0.752 | **1** | 0.392 |
| *CYP75B* | 0.045 | 0.016 | 0.181 | 0.444 | 0.047 | 0.125 | 0.158 | 0.057 | -0.106 | -0.159 | **0.761** | 0.065 | 0.079 | **0.757** | -0.302 | 0.655 | 0.095 | 0.685 | -0.383 | -0.564 | -0.06 | 0.112 | 0.413 | 0.408 | 0.433 | **0.774** | -0.496 | -0.16 | 0.392 | **1** |

**Suppl. Table S3** Irradiance, total dose and ratio between total dose (present study) and the total daily integrated dose (185.5 kJ m^-2^**^*^**) in northern Europe (7 days exposure).

| **UVB irradiance (W m^-2^)** | **Total dose (kJ m^-2^)** | **Ratio (Total/Daily integrated dose)*** |
| --- | --- | --- |
| 0.00 | 0.00 | 0.00 |
| 0.01 | 4.8 | 0.03 |
| 0.23 | 140.3 | 0.8 |
| 0.49 | 293.9 | 1.6 |
| 1.08 | 650.2 | 3.5 |
| 2.04 | 1236.2 | 6.7 |
| 4.18 | 2528.7 | 13.7 |

* Source: UV database of Norwegian Radiation and Nuclear Safety Authority (DSA), <https://github.com/uvnrpa>

**Suppl. Table S4.** No observed effect irradiance (NOEI), no observed effect dose (NOED), lowest observed effect irradiance (LOEI), lowest observed effect dose (LOED, mean of 4 replicates ± SE) and regression coefficient (R^2^) of selected genes in *Lemna minor* after 7days exposure to different irradiances of UVB radiation.

| **Endpoints*** | **NOEI (W m^-2^)** | **NOED (KJ m^-2^)** | **LOEI (W m^-2^)** | **LOED (KJ m^-2^)** |
| --- | --- | --- | --- | --- |
| *SOD* | 0.23 | 140.8 | 0.48 | 293.9 |
| *GPX* | 1.08 | 650.1 | 2.04 | 1233.8 |
| *DRT111* | 0.23 | 140.8 | 0.48 | 293.9 |
| *NDUFV1* | 0.23 | 140.8 | 0.48 | 293.9 |
| *ATPC1* | 0.008 | 4.8 | 0.23 | 140.8 |
| *PK* | 1.08 | 650.1 | 2.04 | 1233.8 |
| *RBSC* | N/A | N/A | N/A | N/A |
| *RAD50* | 0.23 | 140.8 | 0.48 | 293.9 |
| *API5* | 0.48 | 293.9 | 1.08 | 650.1 |
| *ATM* | 0.48 | 293.9 | 1.08 | 650.1 |
| *AMC1* | 0.23 | 140.8 | 0.48 | 293.9 |
| *CYC* | 0.23 | 140.8 | 0.48 | 293.9 |
| *CHL2* | 1.08 | 650.1 | 2.04 | 1233.8 |
| *PSY2* | 1.08 | 650.1 | 2.04 | 1233.8 |
| *CYP75B* | 0.23 | 140.8 | 0.48 | 293.9 |
| *PSBA* | 0.008 | 4.8 | 0.23 | 140.8 |

***** *SOD*, [Superoxide](https://www.sciencedirect.com/topics/earth-and-planetary-sciences/inorganic-peroxide) dismutase; *GPX*, Glutathione peroxidase; *DRT111*, DNA damage-repair/toleration protein; *NDUFV1*, NADH dehydrogenase; *ATPC*1, ATP synthase gamma chain; *PK*, pyruvate kinase; *RBSC*, ribulose bisphosphate carboxylase; *Rad50*, DNA repair protein rad50; *API5*, apoptosis inhibitor 5-like; *ATM*, serine threonine-protein kinase; *AMC1*, metacaspase-1; *CYC*, Cytochrome c; *CHL2*, chlorophyllase-2; *PSY2*, phytoene synthase; *CYP75B*, Flavonoid 3’-monoxygenase; *PSBA*, Photosystem II protein D1. N/A, not applicable/achieved.


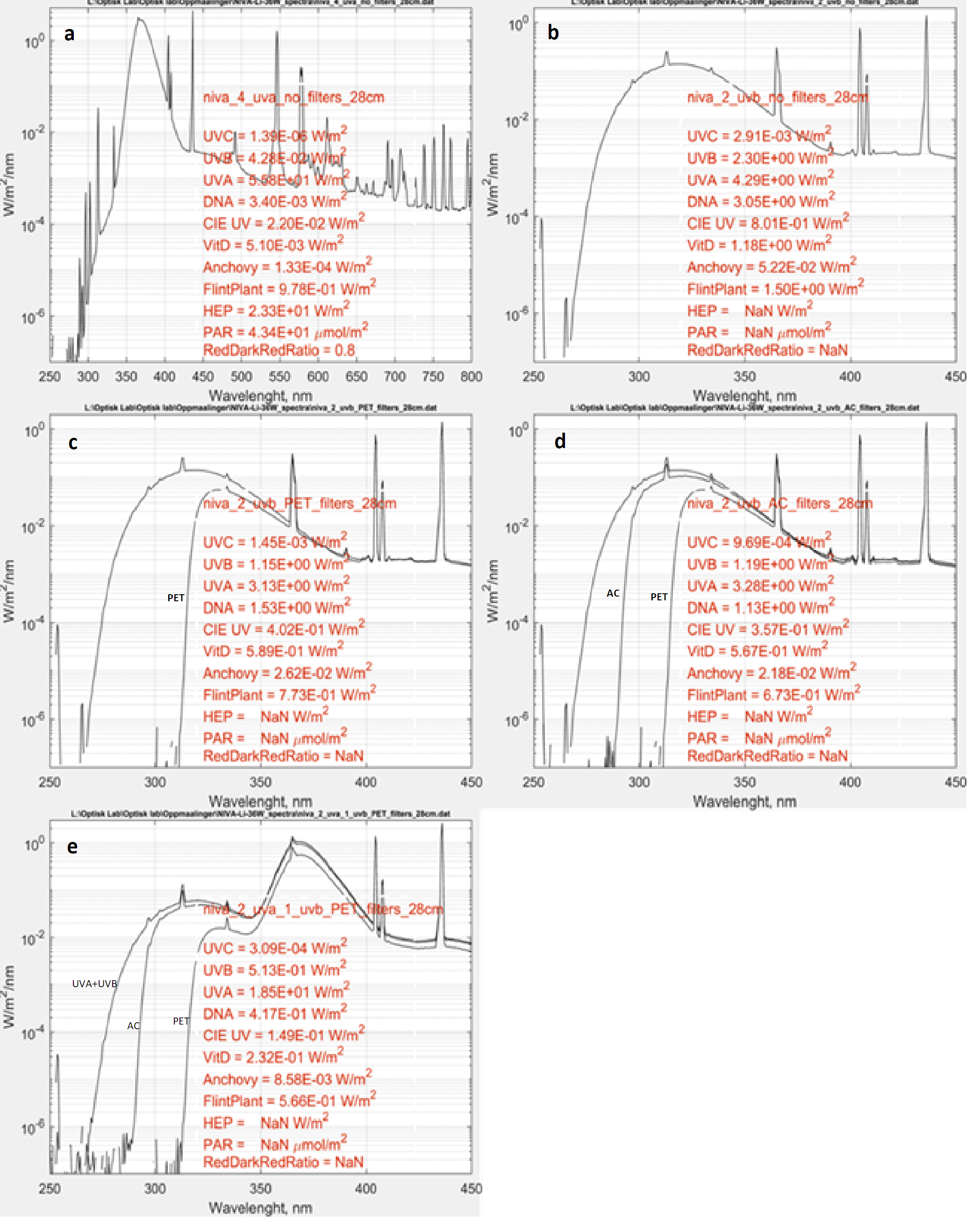


**Suppl. Fig. S1** Spectra of the fluorescent UV tubes with and without filters, **a** UVA tube without filter, **b** UVB tube without filter, **c** UVB tube with polyester foil (PET), **d** UVB tube with cellulose acetate (AC) or polyester foil (PET) and **e** UVA and UVB tube with cellulose acetate (AC) or polyester foil (PET). UVC, irradiance 250 ≤ 𝜆 < 280 nm; UVB, irradiance 280 ≤ 𝜆 < 315 nm; UVA, irradiance 315 ≤ 𝜆 < 400 nm; DNA, the biologically effective irradiance for DNA damage: CIE, the erythemally effective irradiance calculated on basis of the recommendations of the International Commission on Illumination; VitD, the biologically effective irradiance for the production of pre-vitamin D3 in human skin; Ancohovy, the biologically effective irradiance for damage to eggs and larvae of northern anchovy; Flintplant, the biologically effective irradiance for growth responses of plants; HEP, the biologically effective irradiance of hematopoietic porphyria; PAR, photosynthetically active radiation; RedDarkRedratio, ratio between red (650 ≤ 𝜆 ≤ 670 nm) and far red (720 ≤ 𝜆 ≤ 740 nm).


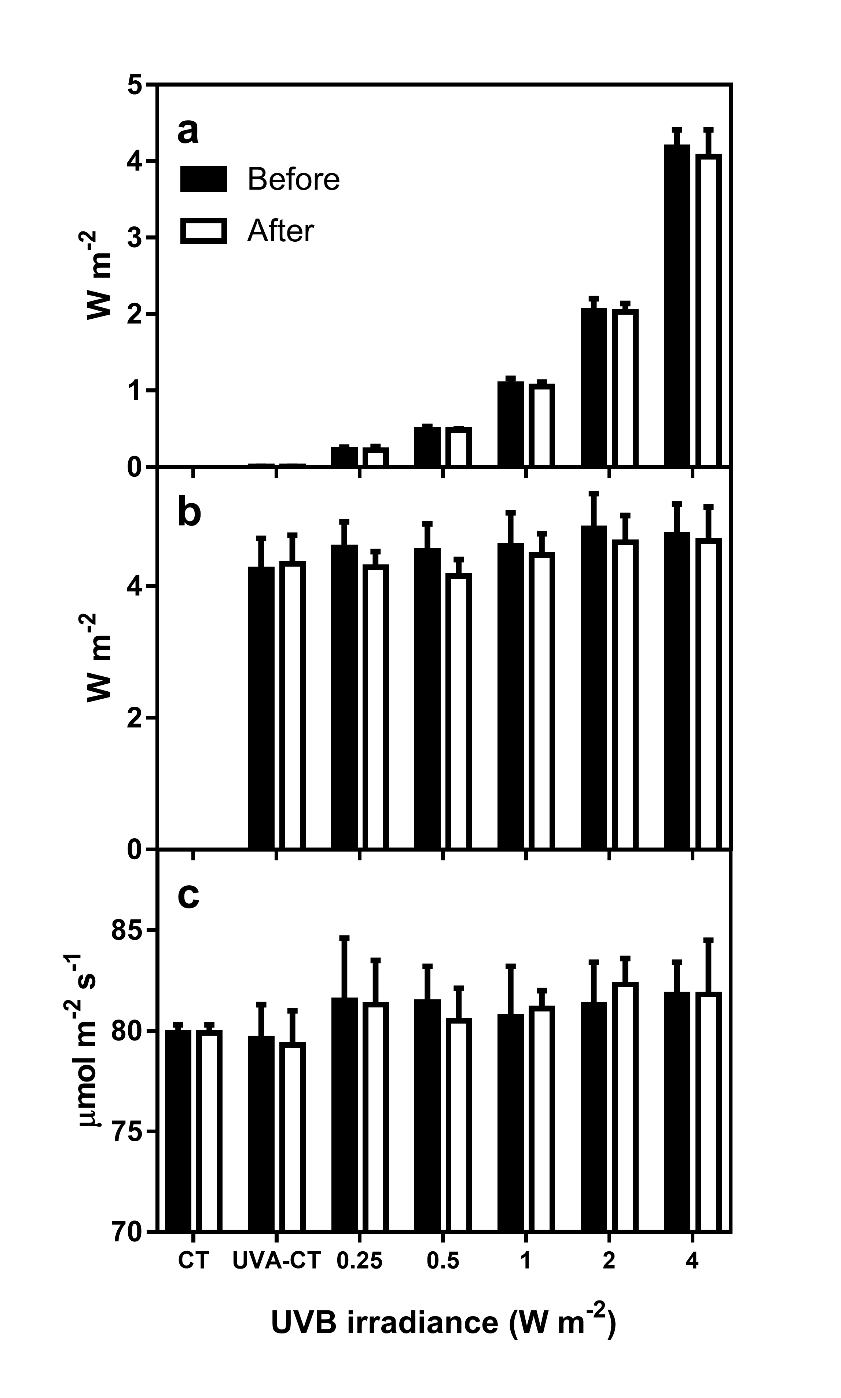


**Suppl. Fig. S2** Intensity of light before and after 7 days’ exposure, **a** UVB, **b** UVA and **c** PAR. (mean of 4 replicates ± SE). CT refers to non-UV control, while UVA-CT refers to the UVA control.


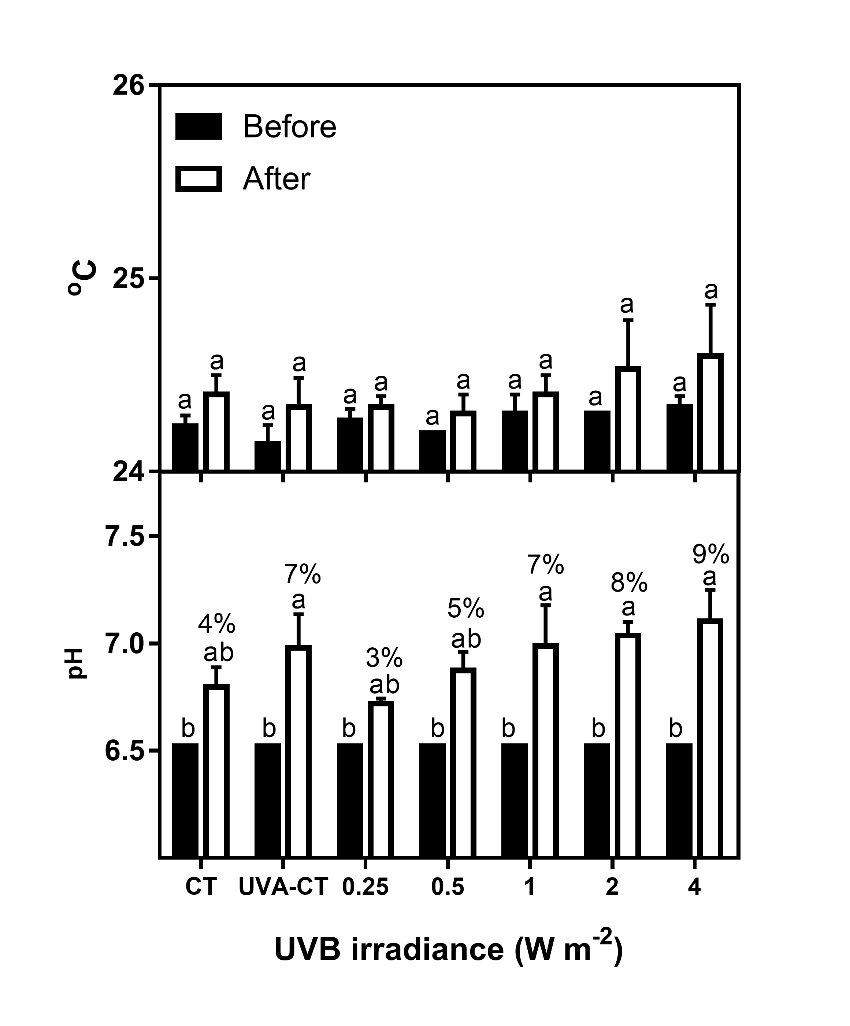


**Suppl. Fig. S3** **a** Temperature and **b** pH before and after 7 days’ exposure to different irradiances of UVB. The letters above the columns indicate differences bewtween groups within a subfigure (mean of 4 replicates ± SE). CT refers to non-UV control, while UVA-CT refers to the UVA control.


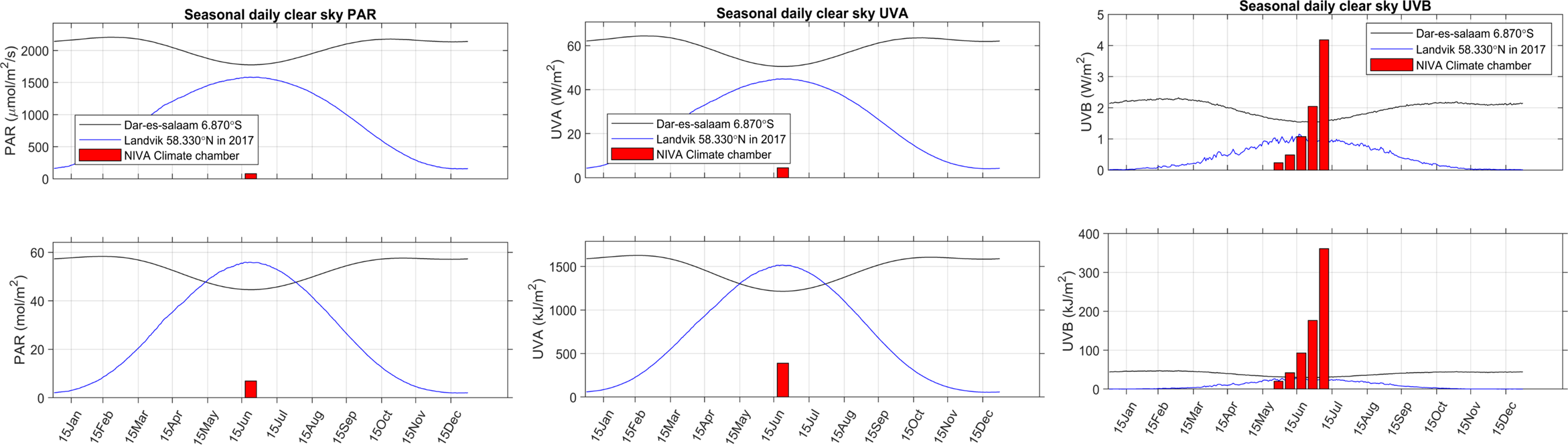


**Suppl. Fig. S4** Levels of PAR, UVA and UVB (Row-1: noon irradiance; Row-2: total dose per day) used in this study compared to ambient, clear sky levels at two sites representing northern Europe (Landvik) and the equatorial region (Dar-es-salaam), with the latitudes provided. Source (Landvik): UV database of the Norwegian Radiation and Nuclear Safety Authority (DSA), <https://github.com/uvnrpa>. Source (Dar-es-salaam): Simulated clear sky levels, applying the radiative transfer software package libRadtran Version 1.7 (Mayer and Kylling, 2005). Model input parameters: solar zenith angle, surface albedo, seasonal mean total ozone extracted from satellite overpass data from the Ozone Monitoring Instrument (OMI) on board the NASA AURA spacecraft (https://avdc.gsfc.nasa.gov/pub/data/satellite/Aura/OMI/V03/L2OVP/OMUVB/[aura_omi_l2ovp_omuvb_v03_dar.es.salaam.txt](https://avdc.gsfc.nasa.gov/pub/data/satellite/Aura/OMI/V03/L2OVP/OMUVB/aura_omi_l2ovp_omuvb_v03_dar.es.salaam.txt)).

**References.**

Mayer, B. and Kylling, A.: Technical note: The libRadtran software package for radiative transfer calculations - description and examples of use, Atmos. Chem. Phys., 5, 1855–1877, https://doi.org/10.5194/acp-5-1855-2005, 2005.
